# Taxonomic re-examination of “*Chloromonas nivalis* (Volvocales, Chlorophyceae) zygotes” from Japan and description of *C*. *muramotoi* sp. nov.

**DOI:** 10.1371/journal.pone.0210986

**Published:** 2019-01-24

**Authors:** Ryo Matsuzaki, Hisayoshi Nozaki, Nozomu Takeuchi, Yoshiaki Hara, Masanobu Kawachi

**Affiliations:** 1 Center for Environmental Biology and Ecosystem Studies, National Institute for Environmental Studies, Onogawa, Tsukuba, Ibaraki, Japan; 2 Department of Biological Sciences, Graduate School of Science, University of Tokyo, Hongo, Bunkyo-ku, Tokyo, Japan; 3 Department of Earth Sciences, Graduate School of Science, Chiba University, Yayoicho, Inage ward, Chiba, Chiba, Japan; 4 Institute of Arts and Sciences, Yamagata University, Kojirakawa, Yamagata, Yamagata, Japan; Donald Danforth Plant Science Center, UNITED STATES

## Abstract

Recent molecular data has strongly suggested that field-collected cysts of snow algae that are morphologically identifiable as the zygotes of *Chloromonas nivalis* are composed of multiple species. Motile vegetative cells, however, have not been directly obtained from these cysts because of the difficulties involved in inducing their germination. Recently, our comparative molecular analyses, using both field-collected and cultured materials, demonstrated that one Japanese lineage of “*C*. *nivalis* zygotes” belongs to *C*. *miwae*. Herein, we examined another Japanese lineage of field-collected “*C*. *nivalis* zygotes” and a new strain originating from Japan. Our molecular data demonstrated that these two different life cycle stages are conspecific, and that they represent a new species that we herein describe as *C*. *muramotoi* sp. nov., based on the vegetative and asexual morphological characteristics of the strain. Multigene phylogenetic analyses showed that this new species was sister to *C*. *miwae*. Scanning electron microscopy demonstrated that the cysts of *C*. *muramotoi* are different from those of *C*. *miwae*, based on the arrangement of the flanges developing on the cell wall.

## Introduction

Snowfields and snowpacks are generally considered extreme environments due to e.g. low temperature, excessive irradiation, and minimal nutrients. However, many species of unicellular microalgae have adapted to such harsh environmental conditions and exclusively thrive within melting snow during spring to summer [1–5]. Blooms of snow-inhabiting microalgae can stain snow green, red, or other colors, and such phenomena are known as “colored snow.” Among snow algae, species belonging to the genus *Chloromonas* Gobi (Volvocales, Chlorophyceae) sensu Pröschold et al. [6] (i.e. *Chloromonadinia* clade [7]) are typical. Most of the species are phylogenetically close and form a robust monophyletic group (subclade 2 of clade A [8,9] or SA clade [10]) within *Chloromonadinia* clade. In snow-inhabiting *Chloromonas* spp., it is known that striking morphological changes from vegetative flagellates to nonmotile cysts/zygotes occur [9,11–15]. Generally, biflagellate vegetative cells are dominant in greenish snow, and nonmotile, dormant cysts or zygotes with a thick cell wall, which accumulate orange carotenoid pigments within the cell, dominate in reddish snow [2–5].

Nonmotile cells or cysts of spindle shape with approximately five to eight flanges on the cell wall are frequently observed within greenish or reddish snowpacks in mid-latitude mountainous areas, as well as in polar regions [1]. According to the species diagnoses from previous studies [12,13], such globally distributed cysts were identified as the zygote stage of *Chloromonas nivalis* (Chodat) Hoham & Mullet. Vegetative cells directly obtained from field-collected cysts identified as *C*. *nivalis* zygotes, however, have not been reported yet. This is possibly due to the difficulty of inducing germination of cysts or zygotes of snow-inhabiting *Chloromonas* species, under controlled laboratory conditions.

Based on short sequences of the large subunit of the RuBisCO (*rbc*L) gene, Muramoto et al. [16] suggested that Japanese “*C*. *nivalis* zygotes” contain at least two independent lineages or species. In addition, they demonstrated the presence of two types of flanges developing on the zygote wall (uniformly straight or sigmate) using scanning electron microscopy (SEM). Correspondence between the two lineages and the two types of flange forms, however, was not revealed. Recently, we established a method for obtaining long sequences of multiple DNA regions from field-collected cysts or zygotes of species of snow-inhabiting *Chloromonas* [17]. By comparing the genetic differences in multiple DNA regions, we showed that field-collected cysts morphologically assignable to *C*. *nivalis* zygotes contained at least four distinct lineages or species; one of which was considered conspecific with *C*. *miwae* (Fukushima) Muramoto et al. Motile vegetative cells corresponding to other lineages of field-collected “*C*. *nivalis* zygotes,” however, have not been observed. In addition, our recent study demonstrated that the North American strain, with vegetative cells morphologically identifiable as *C*. *nivalis*, was phylogenetically separated from the “*C*. *nivalis* zygotes” with molecular data originating from Europe and Japan [18].

Herein, we examined one Japanese lineage of field-collected “*C*. *nivalis* zygotes” and a new strain of snow-inhabiting *Chloromonas* originating from Japan. Our analyses of multiple DNA regions demonstrated that these two different life cycle stages are conspecific, and that they represent a new species that we describe as *C*. *muramotoi* Matsuzaki et al. sp. nov., based on vegetative and asexual characteristics. In addition, we compared the cysts of this new species with those of field-collected cysts of the sister species *C*. *miwae*, using field emission SEM (FE-SEM).

## Materials and methods

### Ethics statement

We collected colored snow from snowpacks in three mountainous areas of Japan: Mt. Gassan in Bandai-Asahi National Park, Mt. Hakkoda in Towada-Hachimantai National Park, and Mt. Tateyama in Chubusangaku National Park. Collection locations and details are shown in S1 Table. No specific permission was required for the present investigation, since collection of snowpacks containing microalgae or other protists from Natural Parks is not legally restricted in Japan. In addition, we confirmed that the field-collected material did not contain protected organisms.

### Observation of field-collected and cultured materials

Colored snow with nonmotile cysts/zygotes of snow-inhabiting *Chloromonas* was collected from summer snowpacks on Mt. Hakkoda, Aomori, Japan, on May 18, 2016 and in Mt. Tateyama, Toyama, Japan, on Jun 12, 2016 (S1 Table). Collection and preservation methods for the colored snow samples were carried out as described previously [19]. Field-collected cysts were identified to species level based on the species descriptions from previous studies [12,13]. Light microscopy (LM) was used to examine the specimens with a BX51 microscope equipped with Nomarski differential interference optics or a CKX41 microscope (Olympus Corp., Tokyo, Japan). For FE-SEM, field-collected cysts were cleaned using a modified sterilizing method [17,20] at room temperature (22–25°C). The samples were subsequently mounted on aluminum stubs with carbon conductive double-faced adhesive tape (Nisshin EM Co. Ltd., Tokyo, Japan) and were air-dried for more than 12 h at 23°C, 25–30% relative humidity. After coating with osmium under the osmium coater HPC-15 (Vacuum Device Corp., Ibaraki, Japan), the cells were observed using a S-4800 field emission scanning electron microscope (Hitachi High-Technologies Corp., Tokyo, Japan). For comparison, we also carried out FE-SEM on *C*. *miwae* cysts within a single snow sample collected from Mt. Gassan, Yamagata, Japan, on Jun 30, 2013, as described above; cysts from the sample were used for the specimen “*C*. *nivalis* zygotes” (Gassan-C) in our previous study [17]. To determine the flange characteristics, we observed 300 cells of “*C*. *nivalis* zygotes” or *C*. *miwae* cysts per field-collected sample under FE-SEM.

*Chloromonas muramotoi* strain HkCl-57 was isolated using the spread plate method [21] from a green snow sample collected from a snowpack in the Hakkoda Botanical Garden of Tohoku University, Mt. Hakkoda, Aomori, Japan, on May 16, 2012 (S1 Table). This strain was deposited as NIES-4284 in the Microbial Culture Collection at the National Institute for Environmental Studies [22]. The culture was maintained on an AF-6 medium (liquid or 1.5% agar slants; see [22]) at 5°C, with a light:dark cycle of 14:10 h under cool-white light-emitting diodes (color temperature = 5000 K) at 35–90 μmol m^−2^ s^−1^. Light and epifluorescence microscopy and transmission electron microscopy (TEM) of the strain were performed as described previously [18]. The method for inducing sexual reproduction by nitrogen starvation was according to a previous study [23].

### Molecular analysis

The method used for extraction of total DNA from the 50 field-collected cysts or the strain HkCl-57 follows that of previous studies [17,24]. The nucleotide sequences of the nuclear-encoded small and large subunits (SSU and LSU, respectively) of ribosomal DNA (rDNA), internal transcribed spacer 2 (ITS2) region of nuclear rDNA, ATP synthase beta subunit (*atp*B), P700 chlorophyll *a* apoprotein A2 (*psa*B), and *rbc*L genes were determined by direct sequencing of PCR products as described previously [17], but using newly designed specific primers (S2 Table) from the three specimens of field-collected cysts morphologically identifiable as the zygotes of *C*. *nivalis* (Hakkoda-Green, Tateyama-Green, and Tateyama-Orange; S1–S3 Figs) and the strain HkCl-57. Since the direct sequencing methodology for the DNA samples extracted from the Japanese cysts resulted in unambiguous data, we did not clone the PCR products.

For the multigene phylogenetic analysis, we used the 31 operational taxonomic units (OTUs) examined in a previous study [18], as well as three specimens of 50 isolated “*C*. *nivalis* zygotes” and *C*. *muramotoi* strain HkCl-57 (S3 Table); all belonging to the genus *Chloromonas* sensu Pröschold et al. [6] or *Chloromonadinia* clade [7]. Since the phylogenetic relationships within the monophyletic group composed entirely of snow species (corresponding to SA clade [10]) were analyzed, we treated the 13 strains that were representatives of the sister group of the SA clade [8–10,25] as the outgroup (S3 Table). The sequences of SSU and LSU rDNA, *atp*B, and *psa*B genes from the OTUs were aligned to the 5,497 base pairs as described previously [25–27]. The resulting data matrix was subjected to Bayesian inference (BI), maximum likelihood (ML), maximum parsimony (MP), and neighbor-joining (NJ) analyses, following methods described in a previous study [18]. Since unusual *rbc*L gene substitutions in *Chloromonas* are common and may result in artefacts [17,26,28], we did not concatenate the *rbc*L gene sequences with the data matrix.

For comparison of the previously published sequence data of field-collected cysts identified as *C*. *nivalis* zygotes, we performed single-gene phylogenetic analyses with wide taxon sampling using *rbc*L gene sequences as described above. Additional OTUs were selected according to previous studies [16,29,30] and are shown in S3 Table. The substitution models for each phylogenetic analysis are described in S4 Table. The data matrices used in this study are available from TreeBASE [31] (matrix accession number, S23486). Methods for annotation and prediction of the secondary structure of the nuclear rDNA ITS2 region follow those described in a previous study [27]. For detecting compensatory base changes (CBCs), the ITS2 sequences were aligned based on a sequence-structure analysis [32] using 4SALE [33,34].

### Nomenclature

The electronic version of this article in Portable Document Format (PDF) in a work with an ISSN or ISBN will represent a published work according to the International Code of Nomenclature for algae, fungi, and plants (Shenzhen Code) (https://www.iapt-taxon.org/nomen/pages/intro/title_page.html), and hence the new names contained in the electronic publication of a PLOS article are effectively published under that Code from the electronic edition alone, so there is no longer any need to provide printed copies.

## Results

### Morphological observation of field-collected cysts

**Fig 1:**
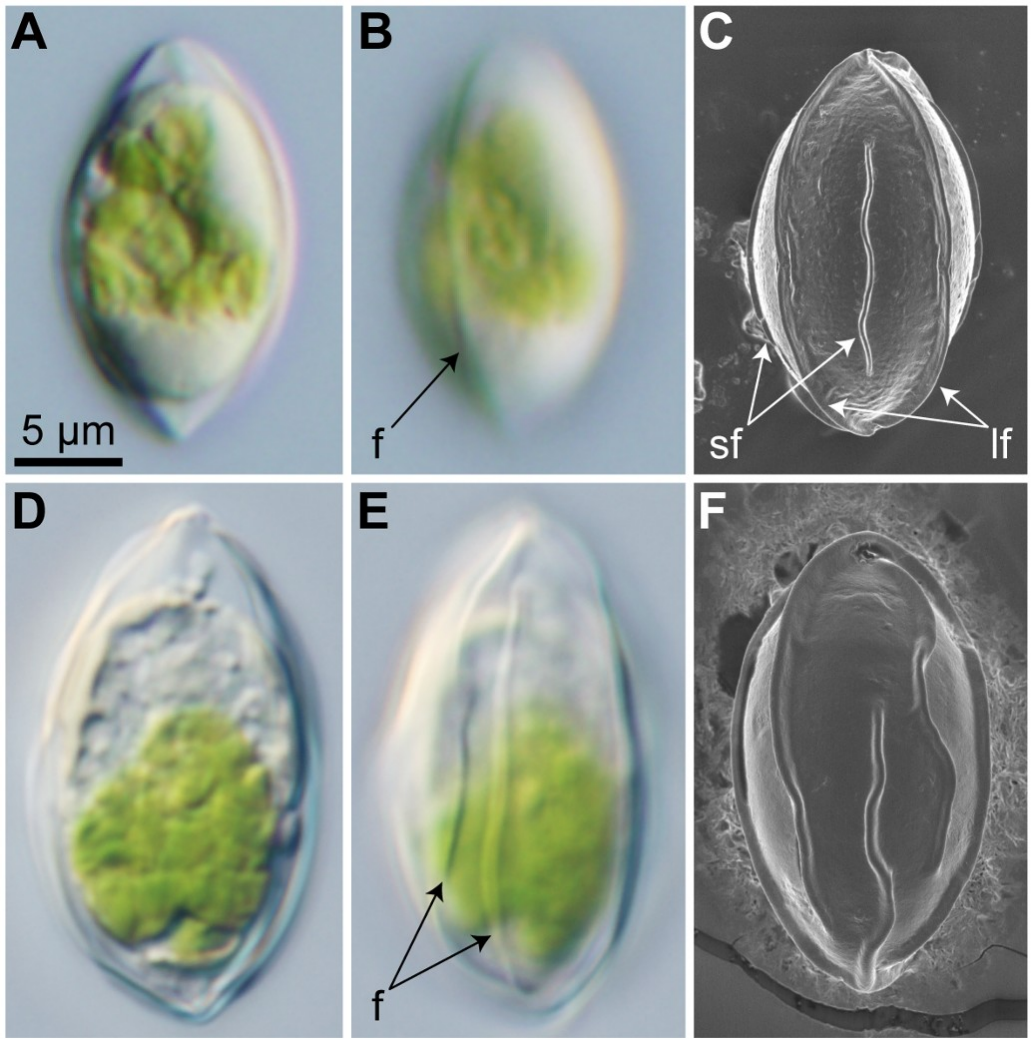
Morphological observation of field-collected cysts/zygotes of snow-inhabiting *Chloromonas*. Identical magnification throughout. For detailed information of collection sites, see S1 Table. (A–C) “*C*. *nivalis* zygotes” from site 160518Hk2G1 in Mt. Hakkoda, Japan. (A, B) Light micrographs. (A) Optical section. (B) Surface view, showing a flange (f). (C) Field emission scanning electron micrograph. Abbreviations: lf, long flange (extending the entire cell length); sf, short flange (reaching neither pole of the cell). (D–F) *C*. *miwae* cysts from site 130630Gs4G in Mt. Gassan, Japan. (D, E) Light micrographs. (D) Optical section. (E) Surface view, showing flanges (f). (F) Field emission scanning electron micrograph.

The characteristics of the cysts collected from Mt. Hakkoda and Mt. Tateyama in Japan were observed to be almost identical. They also corresponded to those of the zygotes of *C*. *nivalis* [12,13]. Cells were spindle-shaped or ellipsoid, 9.1–13.4 μm wide and 15.6–22.4 μm long, with several flanges on the wall (Fig 1A and 1B). The cells from Mt. Hakkoda lacked visible accumulations of carotenoid pigments within the protoplast (specimen Hakkoda-Green; S1 Fig). Conversely, those from Mt. Tateyama were subdivided into two types based on the presence or absence of a large quantity of carotenoid pigments within the cell. Thus, cells of either type were selected and assigned to a single specimen or OTU for our molecular analyses [specimens Tateyama-Green (S2 Fig) and Tateyama-Orange (S3 Fig), respectively].

The results of the FE-SEM revealed that the field-collected “*C*. *nivalis* zygotes” possessed approximately eight flanges at the equatorial plane. The flanges were straight or slightly undulant and could be classified into two types, either long or short (Fig 1C). In each cell, four long flanges (lf in Fig 1C) reached to both poles of the cell, whereas the remaining four short flanges (sf in Fig 1C) were medially located and extended to neither pole. Each short flange was positioned between two long flanges. Bifurcated flanges were not observed, while segmentation and overlapping of flanges was occasionally observed. The cells with those characteristics occupied 59.3% (178/300) and 82.3% (247/300) of “*C*. *nivalis* zygotes” within the field-collected materials from Mt. Hakkoda and Mt. Tateyama, respectively. Although the size and shape of the cells resembled those of the cysts of *C*. *miwae* (which are morphologically assignable to *C*. *nivalis* zygotes [17]) under LM (Fig 1D and 1E), the regular arrangement of the two types of flanges as shown in Fig 1C was hardly observed (1/300) in *C*. *miwae* cysts under FE-SEM (Fig 1F). The number of flanges was commonly 8–10 at the equatorial plane in the cysts of *C*. *miwae*.

### Molecular phylogenetic analyses

**Fig 2:**
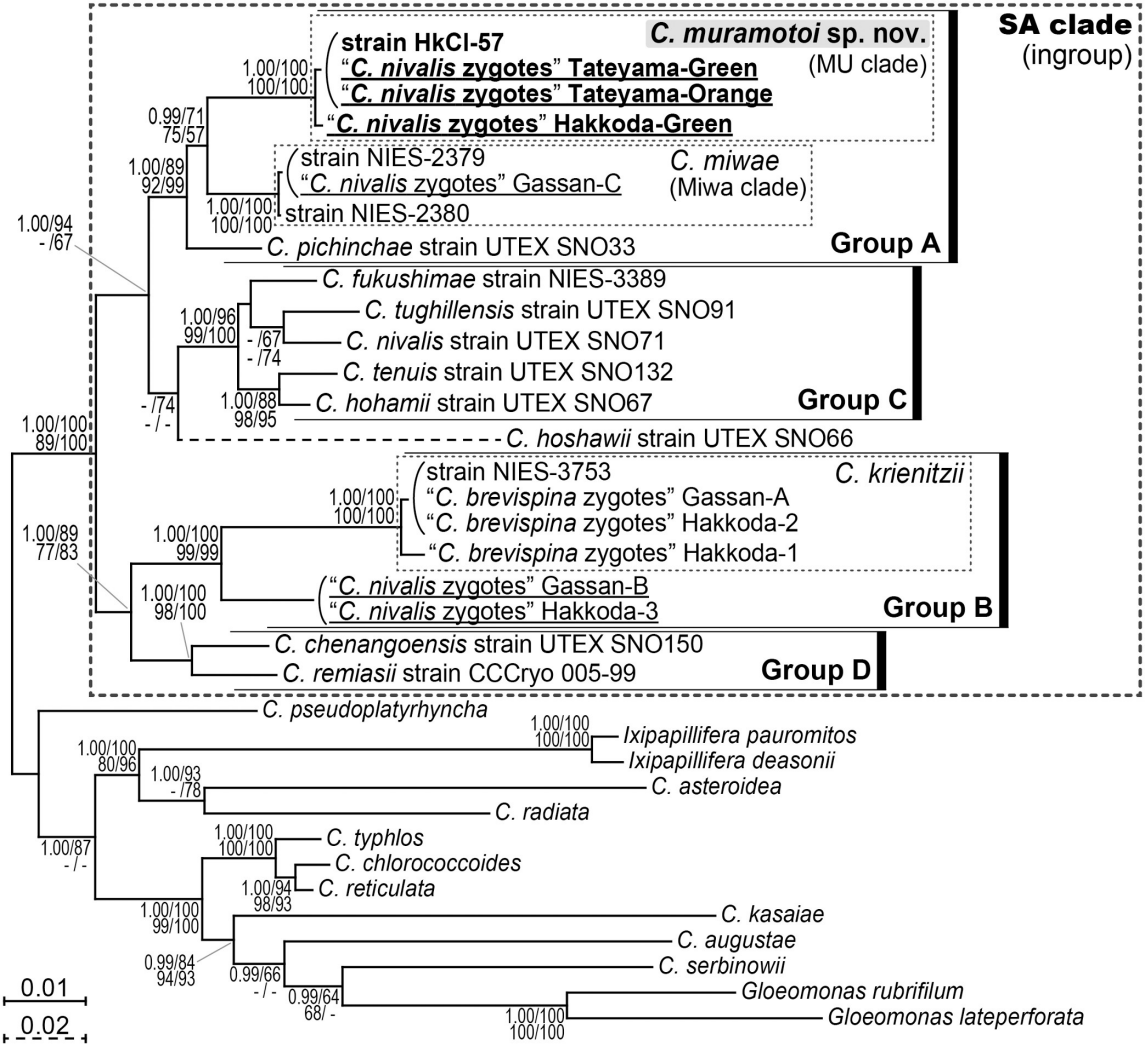
Bayesian phylogenetic tree of snow-inhabiting *Chloromonas* spp. based on 5,497 base pairs from four genes. The small subunit and large subunit of rDNA, and the first and second codon positions of *atp*B and *psa*B genes were partitioned and unlinked (S4 Table). Specimens of field-collected “*C*. *nivalis* zygotes” are underlined. Corresponding posterior probabilities (0.95 or more) are shown at the top left. Numbers shown at the top right, bottom left, and bottom right indicate bootstrap values (50% or more) in the maximum likelihood, maximum parsimony, and neighbor-joining analyses, respectively.

The results of our multigene phylogenetic analysis (Fig 2) essentially corresponded with previous results for species of *Chloromonas* living in snow [18], i.e., four robust monophyletic groups (A–D) and an independent lineage of *C*. *hoshawii* Matsuzaki et al. were resolved. Three Japanese specimens of “*C*. *nivalis* zygotes,” Hakkoda-Green, Tateyama-Green, and Tateyama-Orange, were included within group A, and they formed a small robust subclade (‘MU’ clade) with a Japanese strain HkCl-57, with 1.00 posterior probability (PP) in BI and 100% bootstrap values (BV) in ML, MP, and NJ analyses. Group A also contained another robust subclade (Miwa clade) which was composed of a previously examined Japanese specimen of “*C*. *nivalis* zygotes,” Gassan-C [17], and two Japanese strains of *C*. *miwae* (1.00 PP in BI and 100% BV in ML, MP, and NJ analyses). This subclade was considered as a single species in our recent study [17]. These subclades were sister to each other with moderate statistical support (0.99 PP in BI and 67–75% BV in ML, MP, and NJ analyses). *C*. *pichinchae* Wille strain UTEX SNO33 from North America was the most basal within group A. The other Japanese specimens of “*C*. *nivalis* zygotes” examined previously (Gassan-B and Hakkoda-3 [17]) were positioned within group B. In addition, the *C*. *nivalis* strain UTEX SNO71, originating from North America, was included within group C.

**Fig 3:**
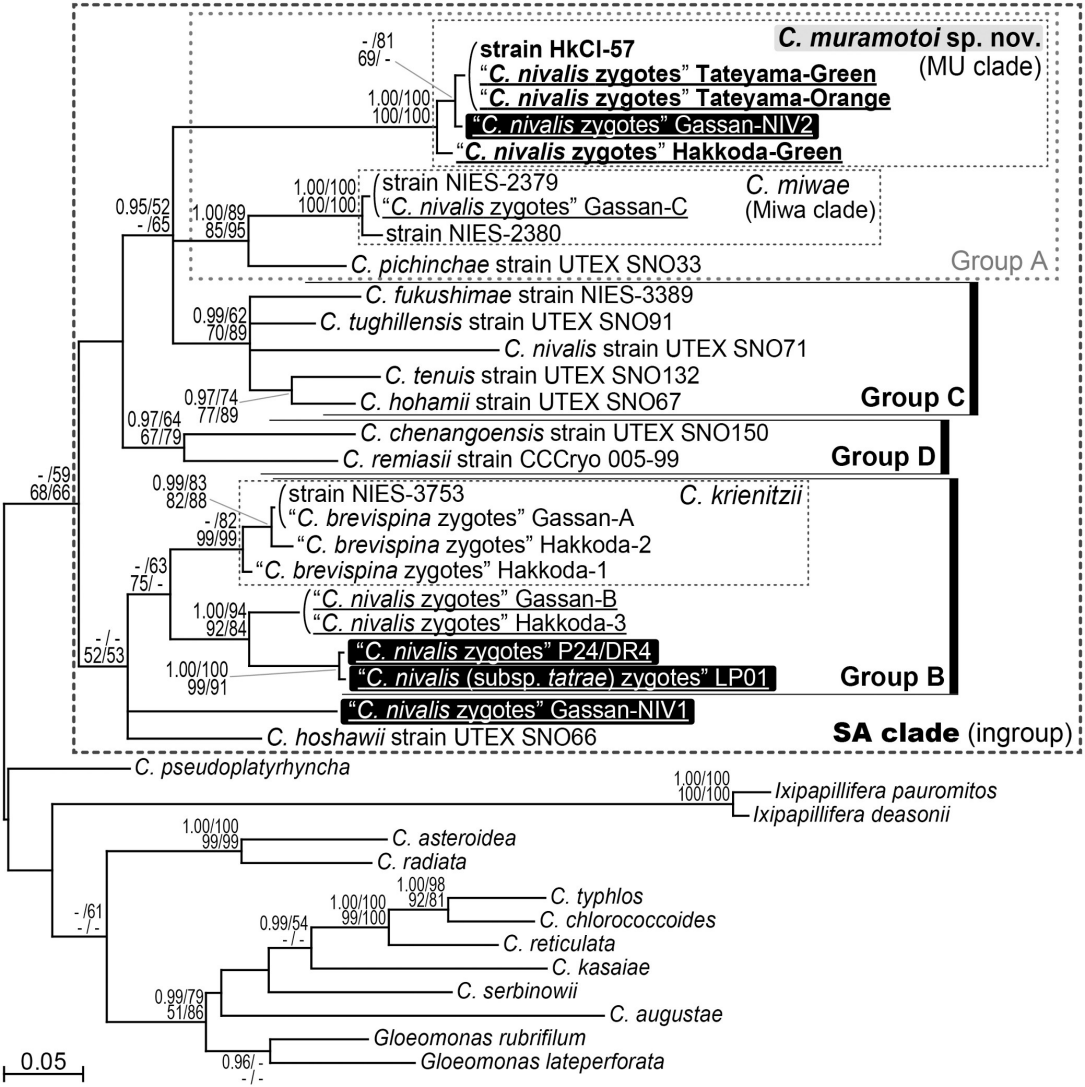
Bayesian phylogenetic tree of snow-inhabiting *Chloromonas* spp. based on 340–1,128 base pairs of *rbc*L. Each codon position was partitioned and unlinked (S4 Table). Specimens of field-collected “*C*. *nivalis* zygotes” are underlined. The operational taxonomic units not included in Fig 2 are highlighted in black. Corresponding posterior probabilities (0.95 or more) are shown at the top left. Numbers shown at the top right, bottom left, and bottom right indicate bootstrap values (50% or more) in the maximum likelihood, maximum parsimony, and neighbor-joining analyses, respectively.

For the *rbc*L-based phylogenetic analysis with wide taxon sampling (Fig 3), groups B–D in the present multigene phylogenetic tree (Fig 2) were reconstructed with lower statistical support values. Conversely, group A in Fig 2 was not resolved in the *rbc*L tree, although the MU and Miwa clades were robustly resolved as shown in Fig 2. This was possibly due to the unusual *rbc*L gene substitution in *Chloromonas* reported by previous studies [17,26,28]. The MU clade, in the *rbc*L-based tree, contained a previously examined Japanese specimen of “*C*. *nivalis* zygotes,” Gassan-NIV2 [16], in addition to the three specimens of “*C*. *nivalis* zygotes” (Hakkoda-Green, Tateyama-Green, and Tateyama-Orange) and the strain HkCl-57 (with 1.00 PP in BI and 100% BV in ML, MP, and NJ analyses). This robust subclade was phylogenetically separated from other specimens of “*C*. *nivalis* zygotes” collected from Europe (LP01 [30] and P24/DR4 [29,30] within group B) as well as those from Japan (Gassan-B and Hakkoda-3 [17] within group B, Gassan-C [17] within the Miwa clade in group A, and Gassan-NIV1 [16] located just outside of group B).

### Comparative analyses of internal transcribed spacer 2

**Fig 4:**
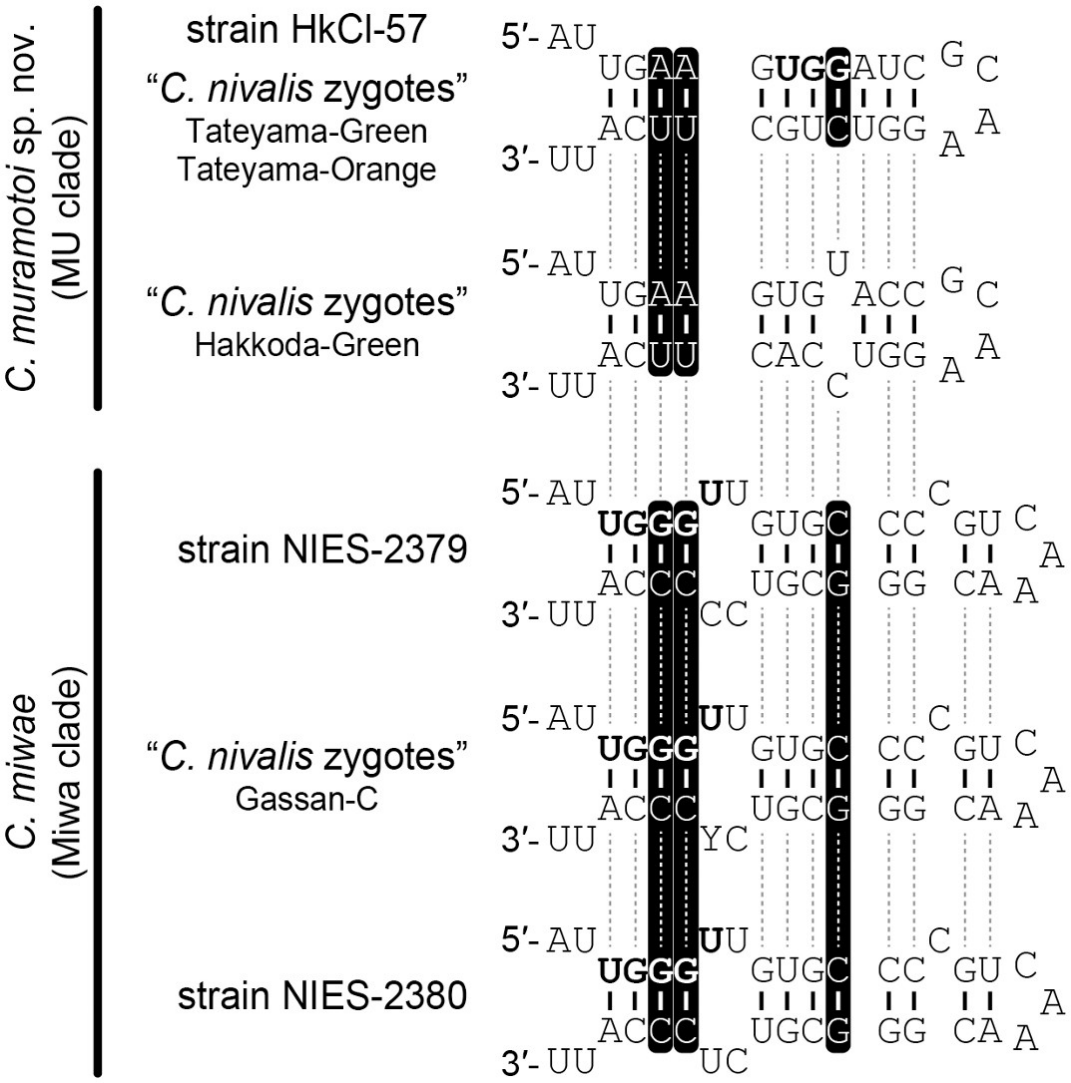
Comparison of the tip of internal transcribed spacer 2 (ITS2) helix III between MU and Miwa clades. For the complete ITS2 secondary structures within the MU clade (= *C*. *muramotoi* sp. nov.), see S4 Fig. Secondary structures of ITS2 within the Miwa clade (= *Chloromonas miwae*) are based on previous results [17]. Black backgrounds indicate compensatory base changes between the two clades. Boldfaces marks the YGGY motif.

Within the MU clade (Fig 2), the number of nucleotide differences in the entire ITS2 region ranged from 0–10 and no CBCs were detected (S4 Fig). Conversely, at least two or three CBCs were detected between the MU and Miwa clades, even near the branch encompassing the YGGY motif in ITS2 helix III, the most conserved region of ITS2 [35] (Fig 4).

### Morphological observation of the *Chloromonas muramotoi* sp. nov. strain HkCl-57

**Fig 5:**
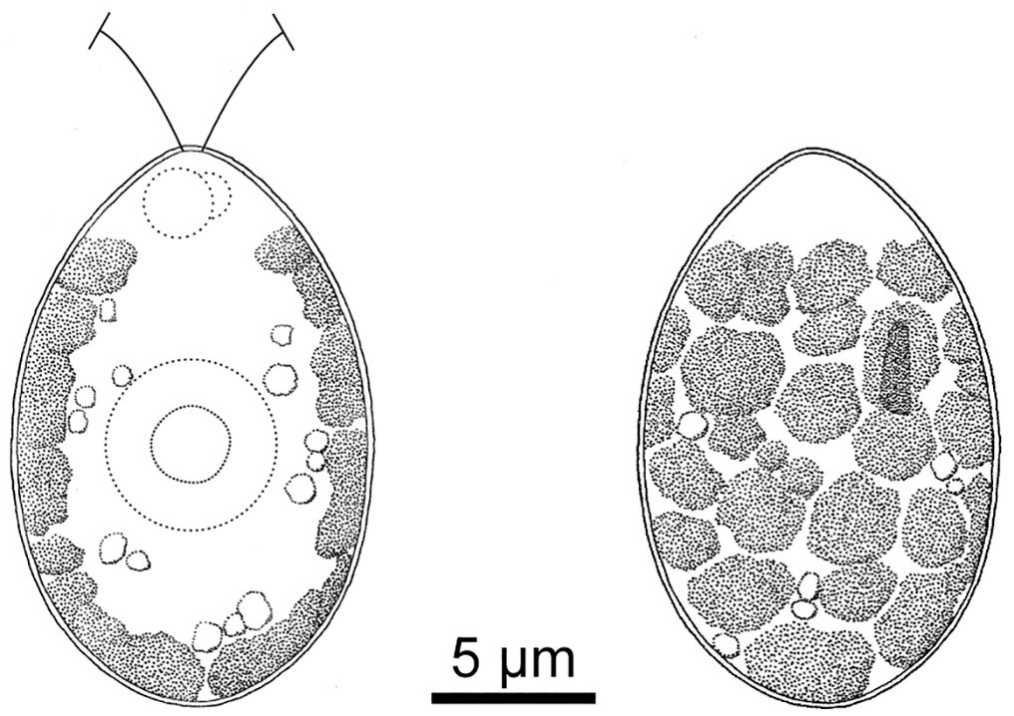
Vegetative cells of *Chloromonas muramotoi* sp. nov.: Line drawings. Anterior end of the cell is arranged upward. Left, optical section. Right, surface view.

**Fig 6:**
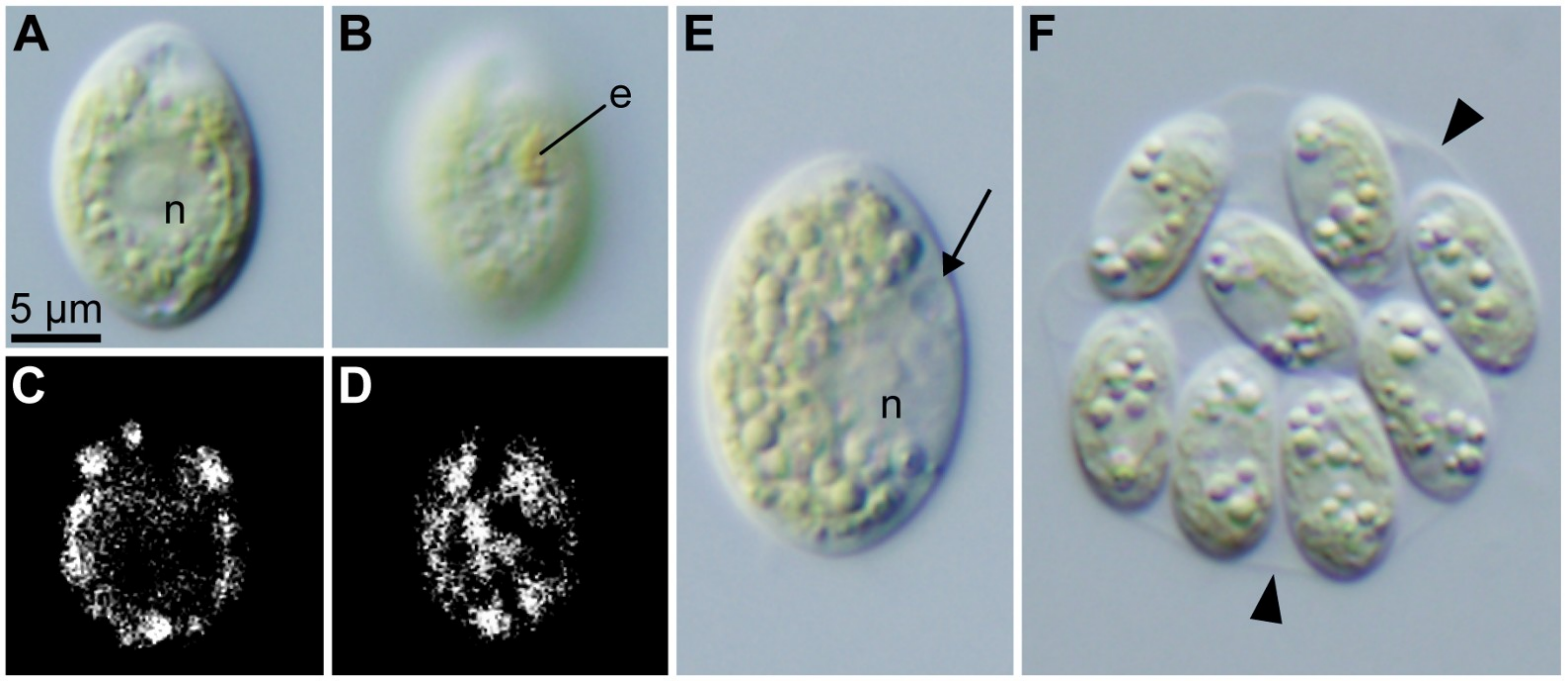
Vegetative and asexual characteristics of *Chloromonas muramotoi* sp. nov. strain HkCl-57: Light micrographs. Identical magnification throughout. Abbreviations: e, eyespot; n, nucleus. (A–D) Vegetative cell. Anterior end of the cell is arranged upward. (A) Optical section. (B) Surface view. (C) Epifluorescence image of (A). (D) Epifluorescence image of (B). (E, F) Asexual reproduction. (E) Just before the first transverse division, showing the position of a parental contractile vacuole (arrow). (F) Autosporangium with eight daughter cells within the parental cell wall (arrowheads).

Under light and fluorescent microscopy, vegetative cells were solitary and ovoid or spindle-shaped with a rounded posterior end, 8.5–13.3 μm wide and 12.3–19.5 μm long (Figs 5 and 6A). Each cell had two equal flagella at the anterior end, a single chloroplast, two contractile vacuoles near the base of the flagella, and a single nucleus, without a prominent anterior papilla (Figs 5 and 6A). The chloroplast was cup-shaped with an eyespot and was lacking pyrenoids (Figs 5 and 6A–6C). The chloroplast was seemingly composed of angular discs, showing irregular incisions in the surface view (Figs 5, 6B and 6D). The eyespot was D-shaped to rod-shaped, positioned in the anterior half to one third of the cell (Figs 5 and 6B). The nucleus was almost spherical, located in the center of the protoplast (Figs 5 and 6A). The flagella were of equal length to the whole cell.

Asexual reproduction was via zoospore formation. Just before the first cell division, the protoplast rotated, and the parental contractile vacuoles moved to the equator of the parent cell (Fig 6E). The protoplast then divided transversely. Generally, two, four, or eight daughter cells were produced within the parental cell wall (Fig 6F). Aggregates of 16 or more cells resulting in repeated division of daughter cells within the parental cell wall [15,18,27] were not produced even in the two-month-old cultures. Sexual reproduction was not observed in the culture even under nitrogen starvation; nor was the production of cysts. Cells of *C*. *muramotoi* did not grow at 20°C after cultivation for 2 weeks, as described in previous reports of other species of snow-inhabiting *Chloromonas* [17,18,27,36].

**Fig 7:**
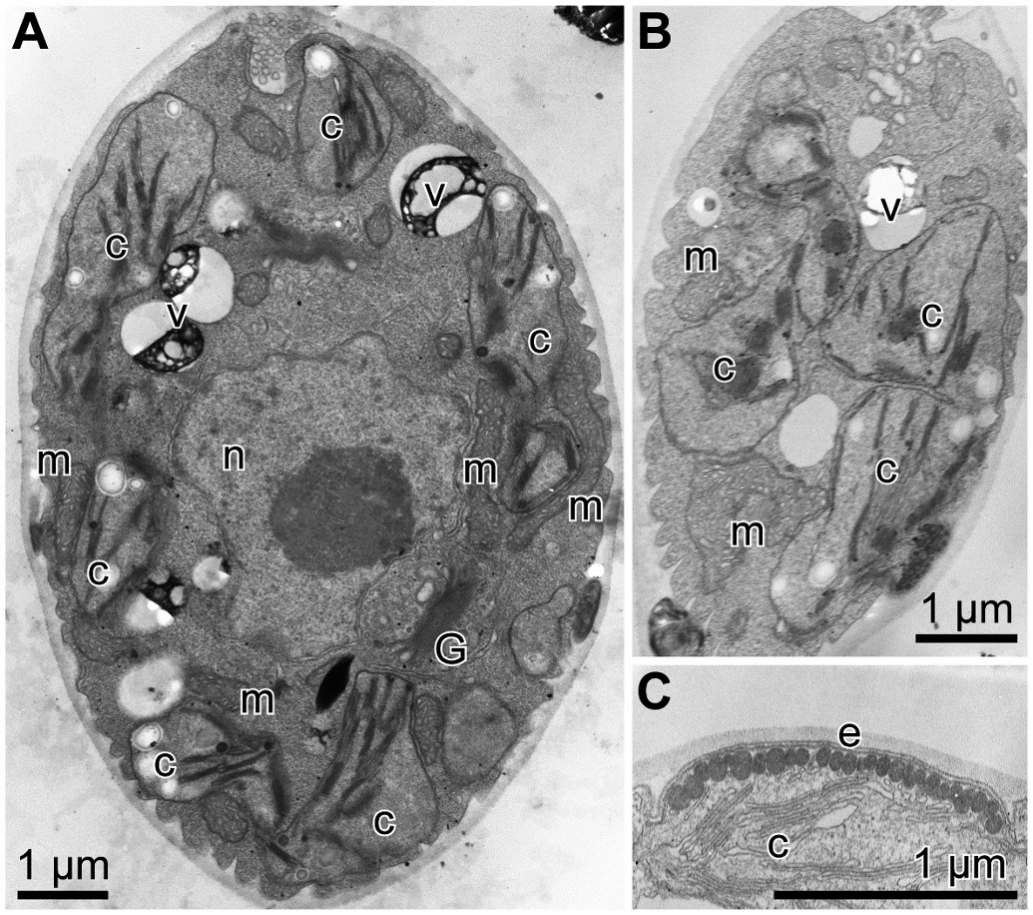
Vegetative cells of *Chloromonas muramotoi* sp. nov. strain HkCl-57: Transmission electron micrographs. Abbreviations: c, chloroplast; e, eyespot; G, Golgi body; m, mitochondrion; n, nucleus; v, vacuole with crystalline content. (A) Longitudinal cell section. (B) Tangential cell section, showing angular chloroplast profiles. (C) Eyespot composed of a single layer of electron-dense globules.

Examination by TEM showed that each cell had a cup-shaped chloroplast without pyrenoid matrices and a centrally located nucleus (Fig 7A). Mitochondria and Golgi bodies were located between the nucleus and chloroplast. In addition, several mitochondria were also recognized near the surface region of the cell, surrounded by chloroplast profiles. As in other snow *Chloromonas* species, small vacuoles with crystalline content were observed in the cytoplasm (e.g. [18]). In the tangential sections of the cell, the chloroplast profiles were almost angular (Fig 7B), corresponding with the LM results (Fig 6B and 6D). The eyespot was composed of a single layer of electron-dense globules (Fig 7C).

## Discussion

The multigene phylogenetic tree presented herein shows that the three Japanese specimens of “*C*. *nivalis* zygotes,” Hakkoda-Green, Tateyama-Green, and Tateyama-Orange, are closely related to the *C*. *muramotoi* strain HkCl-57. This species lacks pyrenoids within the chloroplast, even when examined under TEM, and phylogenetically belongs to *Chloromonadinia* clade, corresponding to both traditional [37,38] and phylogenetically revised [6] generic diagnoses of *Chloromonas*. Among snow-inhabiting *Chloromonas* species, vegetative cells of *C*. *muramotoi* resemble those of *C*. *brevispina* (F.E. Fritsch) Hoham et al. in having ovoid cell shapes without a prominent anterior papilla [14]. In addition, vegetative cells of *C*. *alpina* Wille, *C*. *miwae*, and *C*. *pichinchae* that lack a prominent anterior papilla are sometimes ovoid or elongate-ovoid [11,19,27,39] and are similar to those of *C*. *muramotoi*. *C*. *muramotoi*, however, is distinguished from those four snow-inhabiting species by vegetative cell size, chloroplast morphology, presence of an eyespot, and the lack of production of cell aggregates (resulting from repeated divisions of daughter cells retained within the parental cell wall [15,18,27]) in culture (Table 1).

**Table 1:** Vegetative morphological characteristics of five snow-inhabiting *Chloromonas* species.

	*C*. *muramotoi* sp. nov.	*C*. *alpina*	*C*. *brevispina*	*C*. *miwae*	*C*. *pichinchae*
Strain(s)	HkCl-57	−	−	NIES-2379, NIES-2380	UTEX SNO33
Cell shape	ovoid or spindle-shaped with a rounded posterior end	ellipsoidal to ovoid	ellipsoidal to ovoid or pyriform	spherical or ovoid	elongate-ovoid to ellipsoidal
Cell width × cell length (μm)	8.5–13.3 × 12.3–19.5	4–7 × 9–12	5–13 × 10–19	10–13 × 9–15	8–15 × 18–26
Chloroplast shape	cup-shaped, seemingly composed of angular discs	parietal, seemingly composed of numerous discoid lobes obviously separated from each other	cup-shaped	cup-shaped	cup-shaped, seemingly composed of angular discs
Eyespot	present	present	absent	Absent	absent
Cell aggregates in culture	not observed	−	−	−	observed
References	present study	[39]	[14]	[19]	[11,27,39]

Although *C*. *muramotoi* was isolated from a green snow sample, a comparison of vegetative morphology with *Chloromonas* species not sampled from snow is still informative. Based on Ettl [37,38], *C*. *muramotoi* resembles two species collected from ponds, *C*. *gutenbrunnensis* Wawrik and *C*. *hyperstigmata* (H. Ettl) H. Ettl & Gerloff, in possessing ovoid vegetative cells and a cup-shaped chloroplast with irregular incisions and in lacking a prominent anterior papilla. *C*. *muramotoi* differs from *C*. *gutenbrunnensis*, however, in its smaller vegetative cells (8.5–13.3 μm wide and 12.3–19.5 μm long vs. up to 23 μm wide and up to 27 μm long) and eyespot positions [anterior half to one third (Figs 5 and 6B) vs. posterior half [40]]. *Chloromonas hyperstigmata* possesses quite a large disc-shaped eyespot (diameter up to one fourth of the cell length [41]), whereas, such a large eyespot has never been observed in *C*. *muramotoi* (Figs 5 and 6B). Thus, *C*. *muramotoi* represents a new morphological species of the genus *Chloromonas*, even after careful verification.

*Chloromonas gutenbrunnensis* was collected from an ice-covered pond in winter [40], and *C*. *hyperstigmata* was sampled from a small pond with many fallen leaves in autumn [41]. Therefore, the two species possibly adapt to low temperatures. Interestingly, the vegetative cells of most of the species within SA clade (which is composed entirely of snow algae; Fig 2) possess a chloroplast seemingly subdivided into several lobes or sections [9,18,27]. This indicates that such the chloroplast morphology might be a survival strategy for low temperatures. For instance, the ratio of surface to volume of the chloroplast is in this case better, and an exchange of metabolites with cytoplasm is probably facilitated. Therefore, the morphological similarity of the chloroplasts between *C*. *muramotoi* and the two species not collected from snow may relate to the physiological adaptation to low temperatures.

Within the MU clade (see Fig 2), the genetic differences in a rapidly evolving DNA region (nuclear rDNA ITS2) were observed to be 0.0–4.1%. These results range from being much smaller than to being almost identical to the intraspecific genetic differences in the same region of mesophilic *Chloromonas reticulata* (Goroschankin) Gobi or *Chlamydomonas reinhardtii* P.A. Dangeard (3.3–4.1%) (S5 Fig). In addition, genetic differences in the nuclear-encoded SSU and LSU rDNA and the chloroplast-encoded *atp*B and *psa*B genes within the MU clade were much smaller than those between snow-inhabiting *Chloromonas hohamii* H.U. Ling & Seppelt and *C*. *tenuis* Matsuzaki & Nozaki, or between mesophilic *C*. *chlorococcoides* (H. Ettl & K. Schwarz) Matsuzaki et al. and *C*. *reticulata* (sister species delineated by morphological and molecular data [27,42]) (S6 Fig). Since the comparison of p-distances in these regions did not show differences that were sufficient for distinguishing between species, we concluded that the MU clade should be treated as a single species, namely, *C*. *muramotoi*. In this case, visible carotenoid accumulation observed in the cysts from Mt. Tateyama (assigned to the specimen Tateyama-Orange; S3 Fig) is considered to be a protective reaction against excessive visible and ultraviolet irradiation (see S1 Table). Although *C*. *muramotoi* was sister to *C*. *miwae* (or the Miwa clade [17,19]) (Fig 2), the separation of the two is supported by the differences in vegetative morphology (Table 1), the presence of at least two or three CBCs in the most conserved region of the ITS2 secondary structures (corresponding with the separation of biological species [43]) (Fig 4), and genetic differences (S6 Fig).

Among the specimens of field-collected “*C*. *nivalis* zygotes” with available molecular data, the Japanese specimen Gassan-NIV2 [16] is robustly positioned within the MU clade (= *C*. *muramotoi*) (Fig 3). The available sequence for the specimen (433 base pairs of *rbc*L) is identical to the region of the *C*. *muramotoi* strain HkCl-57 and the two specimens of field-collected cysts (Tateyama-Green and Tateyama-Orange). In addition, a previous study that reported the molecular data of Gassan-NIV2 also exhibited two types of flange morphologies in Japanese “*C*. *nivalis* zygotes” [16], one of which (straight flanges; fig 3 in [16]) is morphologically similar to the cysts of *C*. *muramotoi* (specimens Hakkoda-Green, Tateyama-Green, and Tateyama-Orange; Fig 1C). Thus, we considered that the specimen Gassan-NIV2 may belong to *C*. *muramotoi*, although the morphological characteristics of the specimen at SEM level are unclear.

The present FE-SEM and molecular data indicate that the cysts of *C*. *muramotoi* (which resemble the zygotes of North American *C*. *nivalis*) possess eight regularly arranged flanges (Fig 1C). Among the field-collected *C*. *nivalis* zygotes previously examined by SEM (e.g. [12,16,29,30,44]), this flange arrangement has not been reported, excluding the Japanese *C*. *nivalis* zygotes (see above; S5 Table). Although the morphological variability of flanges has been treated as intraspecific variation [12,13], our FE-SEM results suggest that the flange arrangement of the cysts of *C*. *muramotoi* could be distinguished from that of its sister species, *C*. *miwae* (Fig 1C and 1F). The result indicates that ultrastructural characteristics of the cysts might be useful taxonomic traits at species level, in snow-inhabiting species of *Chloromonas*.

In addition to field-collected “*C*. *nivalis* zygotes,” recent molecular studies have also indicated the necessity for taxonomic re-examination of field-collected nonmotile cells that have been morphologically identified as the cysts or zygotes of snow-inhabiting *Chloromonas* spp. For instance, part of Japanese cysts assigned to the zygotes of *C*. *brevispina* are actually conspecific with *C*. *krienitzii* [17]. *Scotiella cryophila* Chodat (Chlorococcales, Chlorophyceae) is considered to be the asexual cyst of *C*. *rosae* var. *psychrophila* Hoham et al. [8]; however, field-collected cysts identifiable as *S*. *cryophila* originating from Austrian Alps were phylogenetically separated from the authentic strain of *C*. *rosae* var. *psychrophila* from North America [45]. Moreover, part of nonmotile spherical red cells morphologically identified as the cysts of *Chlamydomonas nivalis* (F.A. Bauer) Wille, one of the most famous snow-inhabiting green algae, also seem to belong to the genus *Chloromonas* [46]. Thus, further taxonomic studies of cultured materials as well as increasing molecular data of field-collected cysts/zygotes of snow algae will be required for better understanding of accurate taxonomy, actual diversity, and distribution of snow-inhabiting *Chloromonas* species.

## Taxonomic treatment

### *Chloromonas muramotoi* Matsuzaki, Nozaki & Kawachi sp. nov.

Vegetative cell solitary, with two flagella, without prominent anterior papilla. Cells ovoid or spindle-shaped with rounded posterior end, 8.5–13.3 μm wide and 12.3–19.5 μm long. Cells with central nucleus and single cup-shaped chloroplast. Chloroplast apparently composed of angular disks, with irregularly incised surface and eyespot, without pyrenoids. Eyespot D-shaped to rod-shaped, positioned in anterior half to one third of the cell. Asexual reproduction by formation of usually two, four, or eight zoospores; protoplast rotation prior to the first cell division. Cell aggregates not observed in culture. Sexual reproduction unknown. Zygotes or cysts spindle-shaped or ellipsoid, 9.1–13.4 μm wide and 15.6–22.4 μm long. Cells with commonly eight flanges attached to cell wall; flanges straight or slightly undulant, subdivided into two types; four long flanges reaching to both poles of the cell, four short flanges reaching neither pole of the cell. Each short flange positioned between two long flanges. Flanges not bifurcated generally but segmented and overlapped occasionally.

Holotype: Specimen TNS-AL-58957 deposited at TNS (National Museum of Nature and Science, Tsukuba, Japan); material consists of resin-embedded vegetative cells from strain HkCl-57.

Strain examined: HkCl-57 (Table 1). This strain was deposited as NIES-4284 at the Microbial Culture Collection at the National Institute for Environmental Studies [22].

Etymology: The species epithet ‘*muramotoi*’ is in honor of Mr. Kyohei Muramoto who was the first to indicate that the Japanese specimens of “*C*. *nivalis* zygotes,” Gassan-NIV2 were a separate species using molecular data [16] (Fig 3).

Type locality: 40°38.82′ N, 140°51.08′ E; the Hakkoda Botanical Garden of Tohoku University, Mt. Hakkoda, Aomori, Japan.

Distribution: 40°39.22′ N, 140°50.97′ E; Mt. Hakkoda, Aomori, Japan. 36°35.78′ N, 137°36.63′ E; Mt. Tateyama, Toyama, Japan. The Yamagata Prefectural Nature Conservation Park, Mt. Gassan, Yamagata, Japan [16].

## Supporting information

S1 Fig Fifty “*Chloromonas nivalis* zygotes” assigned to the specimen Hakkoda-Green. Identical magnification throughout. Scale bar = 10 μm.
(JPG)

S2 Fig Fifty “*Chloromonas nivalis* zygotes” assigned to the specimen Tateyama-Green. Identical magnification throughout. Scale bar = 10 μm.
(JPG)

S3 Fig Fifty “*Chloromonas nivalis* zygotes” assigned to the specimen Tateyama-Orange. Identical magnification throughout. Scale bar = 10 μm.
(JPG)

S4 Fig Secondary structure of nuclear ribosomal DNA internal transcribed spacer 2 (ITS2) transcript of *Chloromonas muramotoi* sp. nov. strain HkCl-57. The 3' end of the 5.8S ribosomal RNA (rRNA) and the 5' end of the large subunit (LSU) rRNA are shown (DDBJ/ENA/GenBank accession number: LC438455). The sequences of this region are identical among the strain (HkCl-57) and the two specimens of field-collected “*C*. *nivalis* zygotes” (Tateyama-Green and Tateyama-Orange; DDBJ/ENA/GenBank accession number: LC438456 and LC438457, respectively). Differences between the strain and the specimen of field-collected “*C*. *nivalis* zygotes” (Hakkoda-Green; DDBJ/ENA/GenBank accession number: LC438458) are described just outside the structure. Note U-U mismatch in helix II (arrowheads) and the YGGY motif on the 5' side near the apex of helix III (boldface), common structural hallmarks of eukaryotic ITS2 secondary structures [47,48].
(TIF)

S5 Fig Nucleotide differences (%) in nuclear ribosomal DNA internal transcribed spacer 2. (1) *Chloromonas muramotoi* strain HkCl-57 vs. a specimen of “*C*. *nivalis* zygotes,” Tateyama-Green. (2) Strain HkCl-57 vs. a specimen of “*C*. *nivalis* zygotes,” Tateyama-Orange. (3) Strain HkCl-57 vs. a specimen of “*C*. *nivalis* zygotes,” Hakkoda-Green, (4) *C*. *reticulata* strains, UTEX 1970 (epitype strain proposed by Pröschold et al. [6]) vs. SAG 26.90. (5) *C*. *reticulata* strains, UTEX 1970 vs. SAG 32.86. (6) *Chlamydomonas reinhardtii* strains, SAG 11-32a (which can be crossed with the strain SAG 11-32b, the epitype strain of this species [49]) vs. NIES-2463.
(TIF)

S6 Fig Nucleotide differences (%) from pairwise comparisons in four genes. Black: nuclear-encoded 1,748 bases of the small subunit (SSU) ribosomal DNA (rDNA). Green: nuclear-encoded 2,017 bases of the large subunit (LSU) rDNA. Red: chloroplast-encoded 1,128 bases of ATP synthase beta subunit gene (*atp*B). Blue: chloroplast-encoded P700 chlorophyll *a* apoprotein A2 gene (*psa*B). Note that the sequences from the two specimens of “*Chloromonas nivalis* zygotes,” Tateyama-Green and Tateyama-Orange, were identical in the regions examined. The nucleotide differences between snow-inhabiting and mesophilic sister species (*C*. *hohamii* vs. *C*. *tenuis* and *C*. *chlorococcoides* vs. *C*. *reticulata*) are according to a previous study [27]. (1) The *C*. *muramotoi* strain HkCl-57 vs. a specimen of “*C*. *nivalis* zygotes,” Tateyama-Green. (2) Strain HkCl-57 vs. a specimen of “*C*. *nivalis* zygotes,” Hakkoda-Green. (3) Strain HkCl-57 vs. the *C*. *miwae* strain NIES-2380. (4) Strain HkCl-57 vs. the *C*. *miwae* strain NIES-2379. (5) The *C*. *hohamii* strain UTEX SNO67 vs. the *C*. *tenuis* strain UTEX SNO132. (6) The *C*. *chlorococcoides* strain SAG 15.82 (authentic strain) vs. the *C*. *reticulata* strain UTEX 1970 (epitype strain proposed by Pröschold et al. [6]).
(TIF)

S1 Table Strain/specimens examined in this study. (DOCX)

S2 Table Primers for amplification and sequencing of the ATP synthase beta subunit and the P700 chlorophyll *a* apoprotein A2 genes. (DOCX)

S3 Table Taxa/specimens/strains used for our molecular analyses (Figs 2 and 3; S6 Fig) and DDBJ/ENA/GenBank accession numbers for the five genes. (DOCX)

S4 Table Substitution models applied to the respective data matrices of the present phylogenetic analyses (Figs 2 and 3). (DOCX)

S5 Table Morphological characteristics of field-collected “*Chloromonas nivalis* zygotes” examined by scanning electron microscopy. (DOCX)
