# Supplementary figures and images for: Taxonomic re-examination of “Chloromonas nivalis (Volvocales, Chlorophyceae) zygotes” from Japan and description of C. muramotoi sp. nov
Source: PLoS One. 2019 Jan 24;14(1):e0210986. doi: 10.1371/journal.pone.0210986 (PMC6345437; doi:10.1371/journal.pone.0210986)

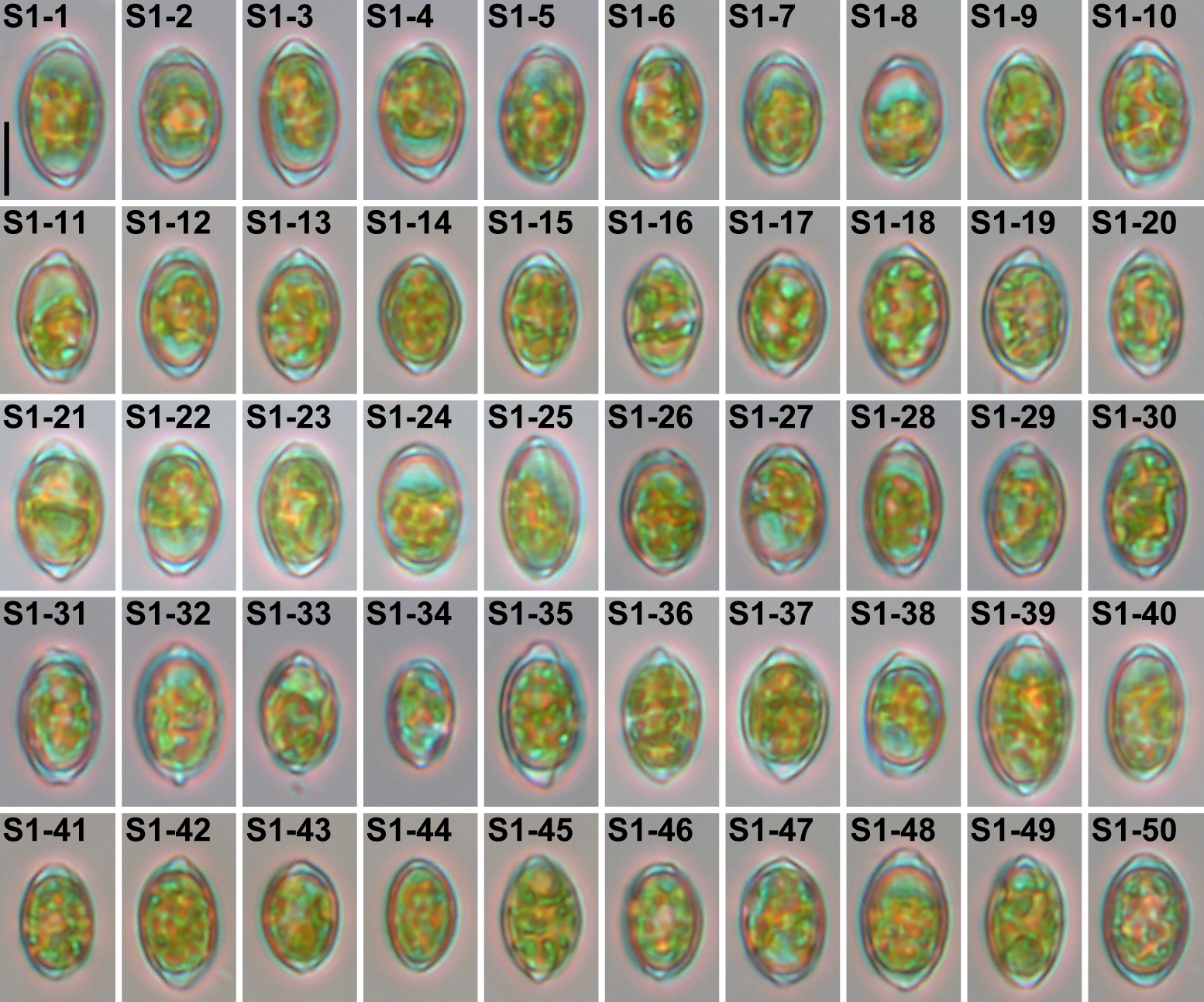

Supplement: S1 Fig — Identical magnification throughout. Scale bar = 10 μm. (JPG) [file pone.0210986.s001.jpg]

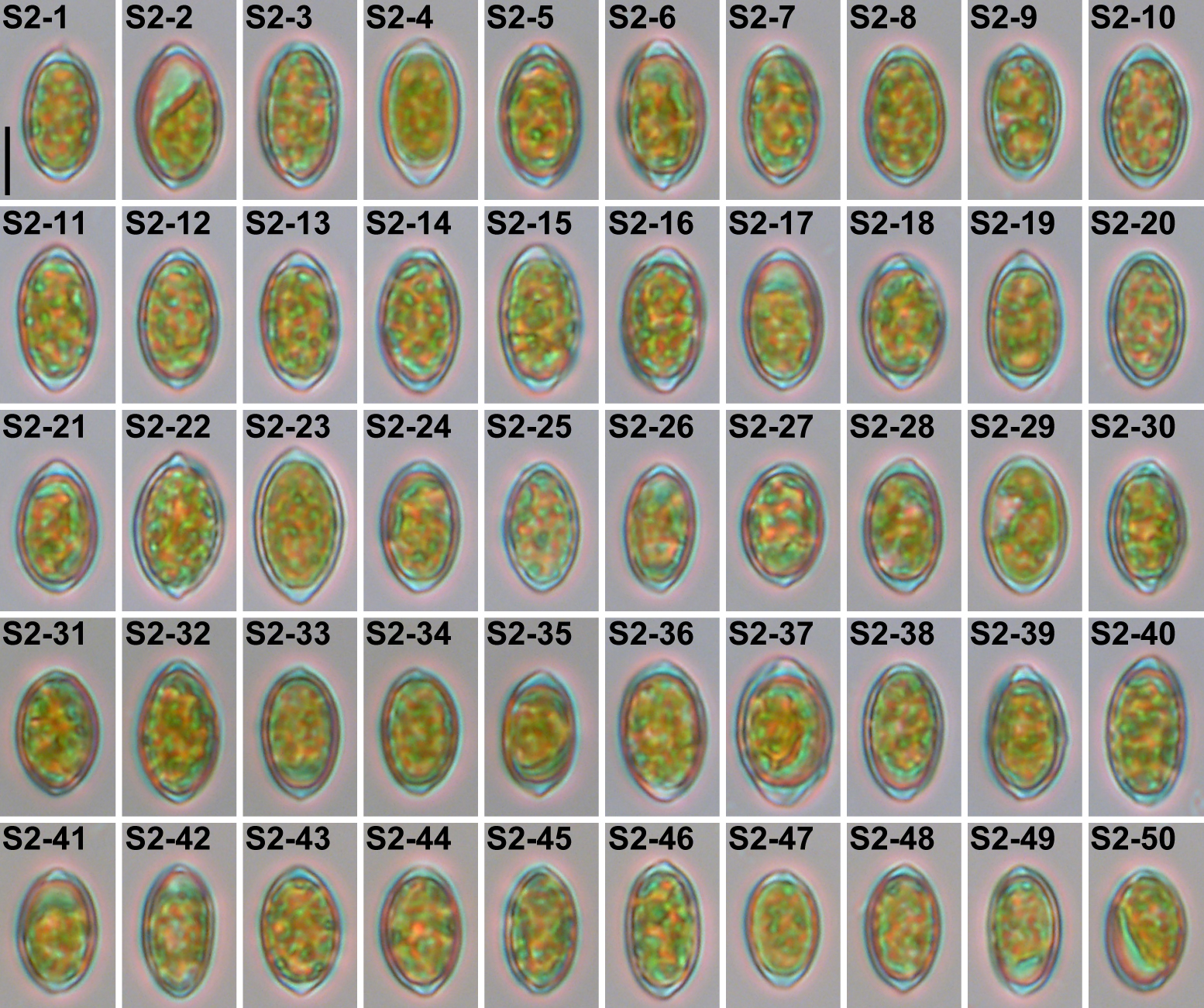

Supplement: S2 Fig — Identical magnification throughout. Scale bar = 10 μm. (JPG) [file pone.0210986.s002.jpg]

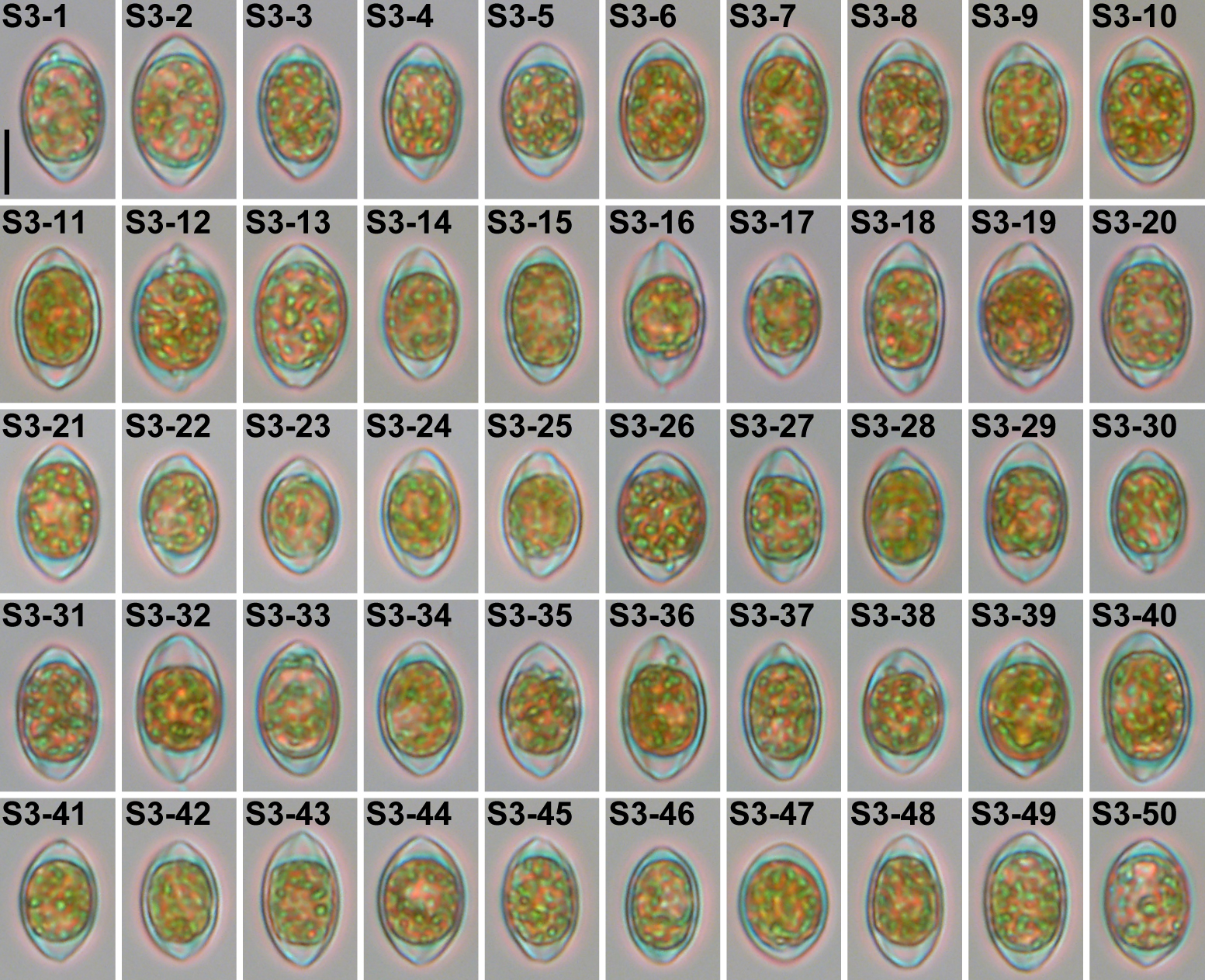

Supplement: S3 Fig — Identical magnification throughout. Scale bar = 10 μm. (JPG) [file pone.0210986.s003.jpg]

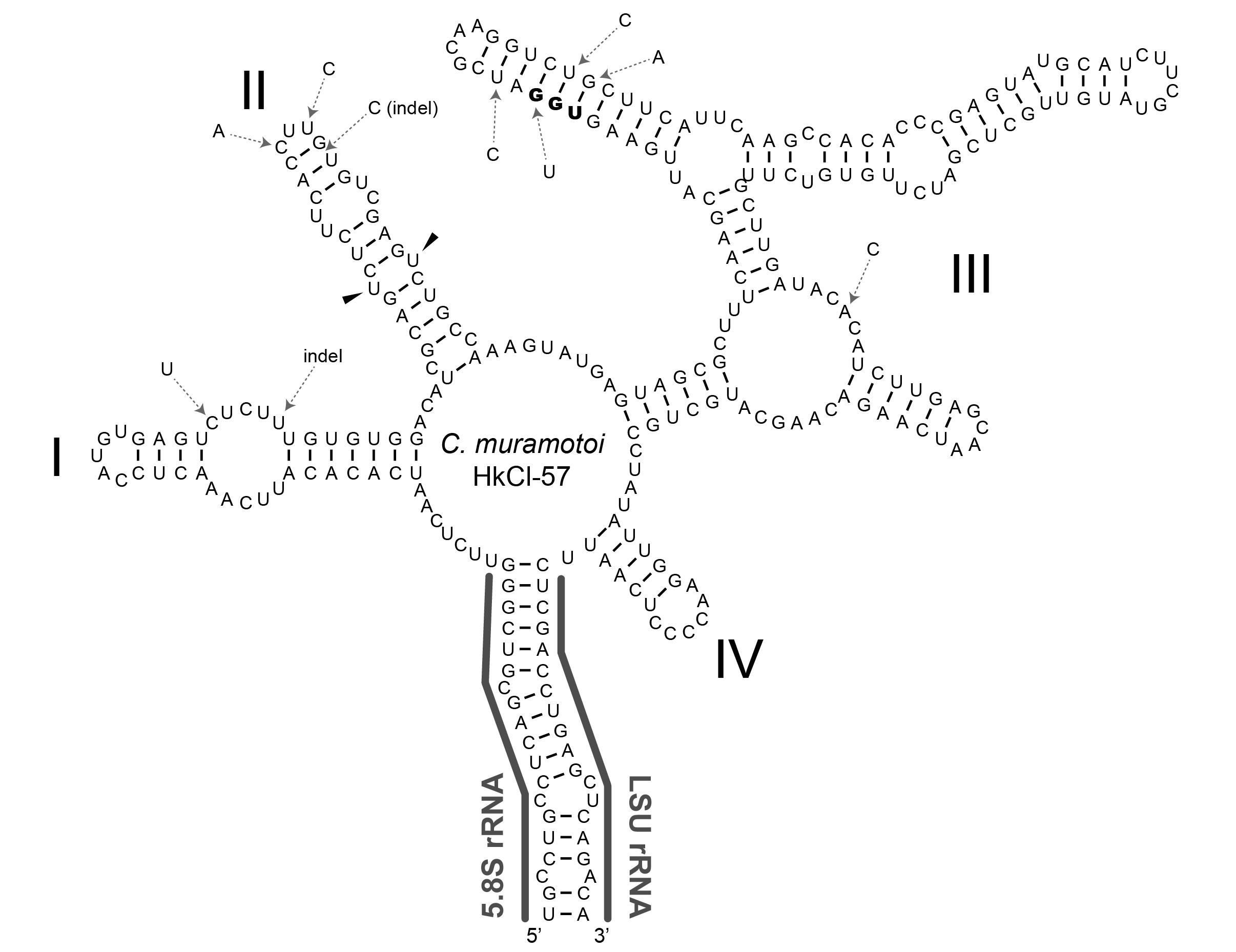

Supplement: S4 Fig — The 3' end of the 5.8S ribosomal RNA (rRNA) and the 5' end of the large subunit (LSU) rRNA are shown (DDBJ/ENA/GenBank accession number: LC438455). The sequences of this region are identical among the strain (HkCl-57) and the two specimens of field-collected “C. nivalis zygotes” (Tateyama-Green and Tateyama-Orange; DDBJ/ENA/GenBank accession number: LC438456 and LC438457, respectively). Differences between the strain and the specimen of field-collected “C. nivalis zygotes” (Hakkoda-Green; DDBJ/ENA/GenBank accession number: LC438458) are described just outside the structure. Note U-U mismatch in helix II (arrowheads) and the YGGY motif on the 5' side near the apex of helix III (boldface), common structural hallmarks of eukaryotic ITS2 secondary structures [47,48]. (TIF) [file pone.0210986.s004.tif]

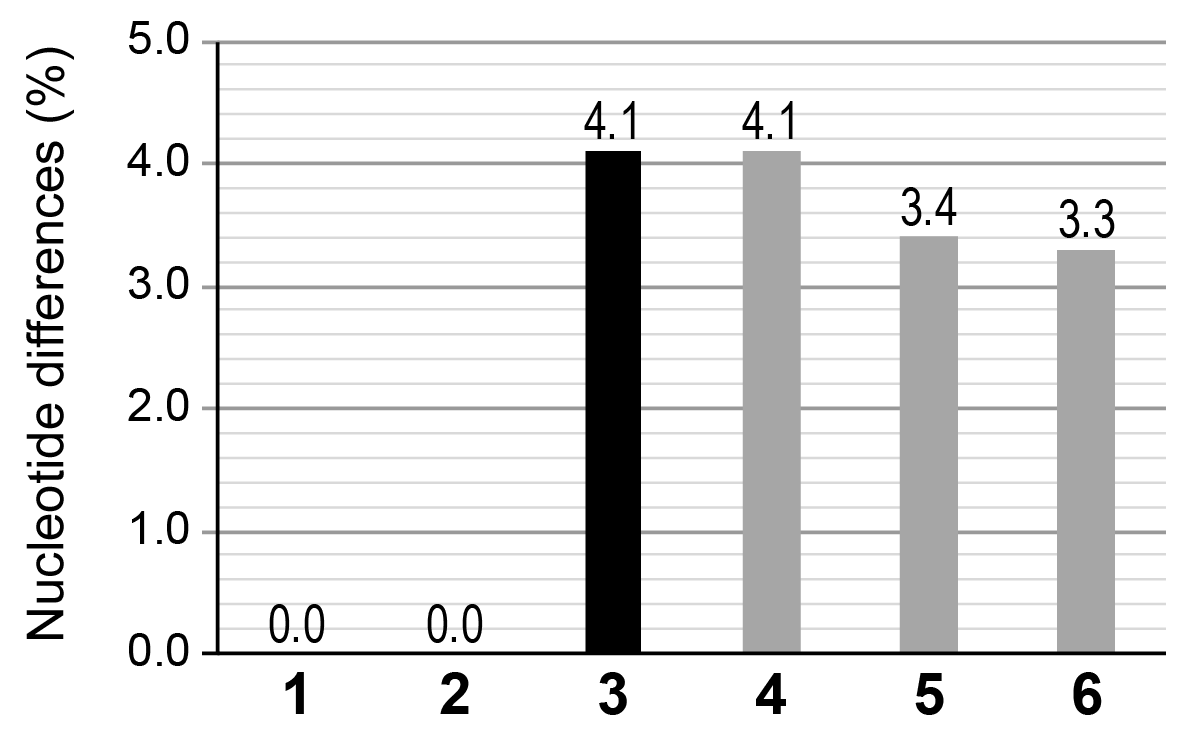

Supplement: S5 Fig — (1) Chloromonas muramotoi strain HkCl-57 vs. a specimen of “C. nivalis zygotes,” Tateyama-Green. (2) Strain HkCl-57 vs. a specimen of “C. nivalis zygotes,” Tateyama-Orange. (3) Strain HkCl-57 vs. a specimen of “C. nivalis zygotes,” Hakkoda-Green, (4) C. reticulata strains, UTEX 1970 (epitype strain proposed by Pröschold et al. [6]) vs. SAG 26.90. (5) C. reticulata strains, UTEX 1970 vs. SAG 32.86. (6) Chlamydomonas reinhardtii strains, SAG 11-32a (which can be crossed with the strain SAG 11-32b, the epitype strain of this species [49]) vs. NIES-2463. (TIF) [file pone.0210986.s005.tif]

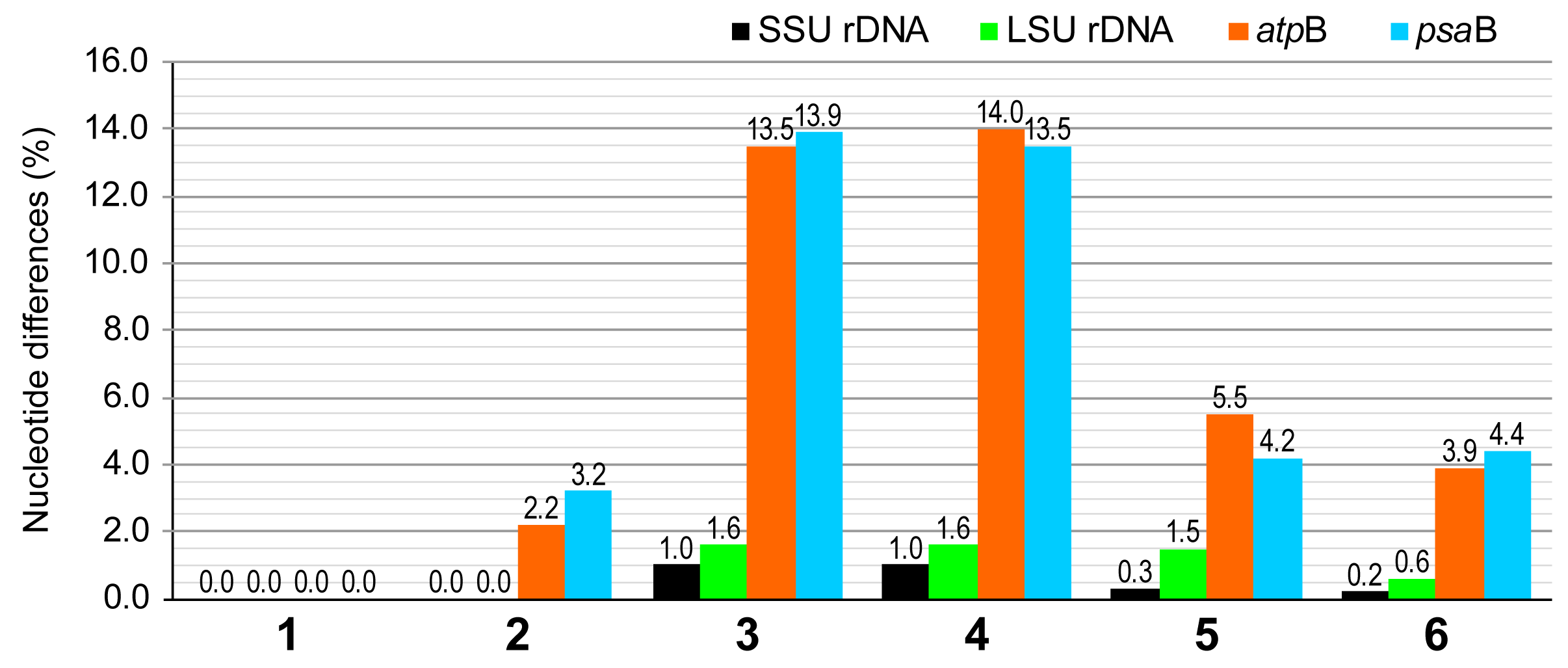

Supplement: S6 Fig — Black: nuclear-encoded 1,748 bases of the small subunit (SSU) ribosomal DNA (rDNA). Green: nuclear-encoded 2,017 bases of the large subunit (LSU) rDNA. Red: chloroplast-encoded 1,128 bases of ATP synthase beta subunit gene (atpB). Blue: chloroplast-encoded P700 chlorophyll a apoprotein A2 gene (psaB). Note that the sequences from the two specimens of “Chloromonas nivalis zygotes,” Tateyama-Green and Tateyama-Orange, were identical in the regions examined. The nucleotide differences between snow-inhabiting and mesophilic sister species (C. hohamii vs. C. tenuis and C. chlorococcoides vs. C. reticulata) are according to a previous study [27]. (1) The C. muramotoi strain HkCl-57 vs. a specimen of “C. nivalis zygotes,” Tateyama-Green. (2) Strain HkCl-57 vs. a specimen of “C. nivalis zygotes,” Hakkoda-Green. (3) Strain HkCl-57 vs. the C. miwae strain NIES-2380. (4) Strain HkCl-57 vs. the C. miwae strain NIES-2379. (5) The C. hohamii strain UTEX SNO67 vs. the C. tenuis strain UTEX SNO132. (6) The C. chlorococcoides strain SAG 15.82 (authentic strain) vs. the C. reticulata strain UTEX 1970 (epitype strain proposed by Pröschold et al. [6]). (TIF) [file pone.0210986.s006.tif]
