# Supplementary material for: Taxonomic re-examination of “Chloromonas nivalis (Volvocales, Chlorophyceae) zygotes” from Japan and description of C. muramotoi sp. nov
Source: PLoS One. 2019 Jan 24;14(1):e0210986. doi: 10.1371/journal.pone.0210986 (PMC6345437; doi:10.1371/journal.pone.0210986)
Supplement: S1 Table — (DOCX) [file pone.0210986.s007.docx]

**S1 Table. Strain/specimens examined in this study.**

| Taxon | Designation | Origin | Elevation | Situation | Collection date |
| --- | --- | --- | --- | --- | --- |
| *Chloromonas muramotoi* sp. nov. | HkCl-57 (=NIES^1^-4284) | Green snow sample collected in Mt. Hakkoda, Aomori, Japan (40°38.83' N, 140°51.08' E) | 883 m | open area with sparse broadleaf trees | May 16, 2012 |
| *Chloromonas miwae* | Gassan-C^2^ | Green snow sample collected from site 130630Gs4G in Mt. Gassan, Yamagata, Japan (38°30.40' N, 139°59.87' E) | 815 m | shaded area by broadleaf trees | Jun 30, 2013 |
| *Chloromonas nivalis*^3^ | Hakkoda-Green | Green snow sample collected from site 160518Hk2G1 in Mt. Hakkoda, Aomori, Japan (40°39.22' N, 140°50.98' E) | 974 m | shaded area by conifers and broadleaf trees | May 18, 2016 |
|  | Tateyama-Green | Red snow sample collected from site 160612Ty2R in Mt. Tateyama, Toyama, Japan (36°34.10' N, 137°35.95' E) | 2,753 m | open, exposed area | Jun 12, 2016 |
|  | Tateyama-Orange | Red snow sample collected from site 160612Ty2R in Mt. Tateyama, Toyama, Japan (36°34.10' N, 137°35.95' E) | 2,753 m | open, exposed area | Jun 12, 2016 |

^1^Microbial Culture Collection at the National Institute for Environmental Studies [1,2].

^2^Specimen of field-collected cysts morphologically assignable to *C. nivalis* zygotes. For species identification, see [3]

^3^Identified based on the species diagnoses from previous studies [4,5].

**References**

1. Kawachi M, Ishimoto M, Mori F, Yumoto K, Sato M, Noël M-H. MCC-NIES. List of Strains, 9th Edition [DVD]. Tsukuba: National Institute for Environmental Studies; 2013.

2. Microbial Culture Collection at National Institute for Environmental Studies [Internet]. Ibaraki: The National Institute for Environmental Studies; c2001 [cited 2018 Oct 17]. Available from: <http://mcc.nies.go.jp/index_en.html>.

3. Matsuzaki R, Kawai-Toyooka H, Hara Y, Nozaki H. Revisiting the taxonomic significance of aplanozygote morphologies of two cosmopolitan snow species of the genus *Chloromonas* (Volvocales, Chlorophyceae). Phycologia. 2015; 54: 491–502. doi: 10.2216/15-33.1.

4. Hoham RW, Mullet JE. The life history and ecology of the snow alga *Chloromonas cryophila* sp. nov. (Chlorophyta, Volvocales). Phycologia. 1977;16: 53–68. doi: 10.2216/i0031-8884-16-1-53.1.

5. Hoham RW, Mullet JE. *Chloromonas nivalis* (Chod.) Hoh. & Mull. comb. nov., and additional comments on the snow alga, *Scotiella*. Phycologia. 1978;17: 106–107. doi: 10.2216/i0031-8884-17-1-106.1.
