# Supplementary material for: Taxonomic re-examination of “Chloromonas nivalis (Volvocales, Chlorophyceae) zygotes” from Japan and description of C. muramotoi sp. nov
Source: PLoS One. 2019 Jan 24;14(1):e0210986. doi: 10.1371/journal.pone.0210986 (PMC6345437; doi:10.1371/journal.pone.0210986)
Supplement: S2 Table — (DOCX) [file pone.0210986.s008.docx]

**S2 Table. Primers for amplification and sequencing of the ATP synthase beta subunit (*atp*B) and the P700 chlorophyll *a* apoprotein A2 (*psa*B) genes.**

| Designation | Position^1^ | Sequence (5′–3′) |
| --- | --- | --- |
| *atp*B |  |  |
| small-nivZF | 201–224 | ACTTCTTGGAGACAATTGTGTTCG |
| Snow-5′R | 584–562^2^ | CCWCCRTGYGCTTTTGCRATATT |
| F2r | 752–770 | TAACWGCWTTAACIATGGC |
| R3r | 890–871^2^ | CCTACIGCWGWIGGCATACG |
| Snow-3′F | 943–965 | TCAACWAAAGAIGGTTCIATYAC |
| small-nivZR | 1409–1387^2^ | GGTAAGCTCTCAAGTTCACCCGC |
|  |  |  |
| *psa*B |  |  |
| Snow-F0r | 247–269 | CATGTICGCCCAATWGCICAYGC |
| small-nivZF | 270–291 | TATTTGGGACCCGCATTTTGGT |
| Snow-R5r | 796–821^2^ | GCCATATCIGTTARCCATAAACTTTG |
| Snow-5′R | 890–869^2^ | ATICCAAARTTIGTRCGRTACA |
| F5r | 989–1010 | TTCATTTYCAAYTAGGITTAGC |
| Snow-3′F | 1372–1394 | GTTTTTGCICAATGGATTCAAGC |
| small-nivZR | 1715–1692^2^ | GCTGAAATATCACATGTTCCACCG |

^1^Coordinate numbers from the *atp*B or *psa*B genes of *Chlorella vulgaris* Beijerinck [1].

^2^Reverse primer.

**Reference**

1. Wakasugi T, Nagai T, Kapoor M, Sugita M, Ito M, Ito S, et al. Complete nucleotide sequence of the chloroplast genome from the green alga *Chlorella vulgaris*: The existence of genes possibly involved in chloroplast division. Proc Natl Acad Sci U S A. 1997;94: 5967–5972. PubMed PMID: 9159184; PubMed Central PMCID: PMC20890.
