# Supplementary material for: Taxonomic re-examination of “Chloromonas nivalis (Volvocales, Chlorophyceae) zygotes” from Japan and description of C. muramotoi sp. nov
Source: PLoS One. 2019 Jan 24;14(1):e0210986. doi: 10.1371/journal.pone.0210986 (PMC6345437; doi:10.1371/journal.pone.0210986)
Supplement: S3 Table — (DOCX) [file pone.0210986.s009.docx]

**S3 Table. Taxa/specimens/strains used for our molecular analyses (Figs 2 and 3; S6 Fig) and DDBJ/ENA/GenBank accession numbers for the five genes.**

| Taxon | Specimen/strain  Designation | Accession number | | | | |
| --- | --- | --- | --- | --- | --- | --- |
|  |  | SSU rDNA | LSU rDNA | *atp*B | *psa*B | *rbc*L |
| Ingroup | | | | | | |
| *C. brevispina* | Gassan-A^1^ | LC012709 | LC060471 | LC012717 | LC012725 | LC012733 |
|  | Hakkoda-1^2^ | LC012710 | LC060472 | LC012718 | LC012726 | LC012734 |
|  | Hakkoda-2^2^ | LC012711 | LC060473 | LC012719 | LC012727 | LC012735 |
| *C. chenangoensis* | UTEX^3^ SNO150  (authentic) | AB906341 | LC360468 | AB906360 | AB906371 | LC012736 |
| *C. fukushimae* | NIES^4^-3389  (authentic) | AB906342 | AB906352 | AB906361 | AB906372 | LC012738 |
| *C. hohamii* | UTEX SNO67 | AB906344 | AB906354 | AB906363 | AB906374 | AB434265,  LC012742 |
| *C. hoshawii* | UTEX SNO66  (authentic) | AB906345 | LC360469 | AB906364 | AB906375 | AB434272 |
| *C. krienitzii* | NIES-3753  (authentic) | LC012712^4^ | LC060474 | LC012720^4^ | LC012728^4^ | LC012740^4^ |
| *C. miwae* | NIES-2379 | AB906350 | LC060476 | AB906369 | AB906380 | AB434271 |
|  | NIES-2380 | AB906351 | LC060477 | AB906370 | AB906381 | AB434270 |
| *C. muramotoi* sp. nov. | HkCl-57^5^  (= NIEX-4284) | LC438435^6^ | LC438439^6^ | LC438443^6^ | LC438447^6^ | LC438451^6^ |
| *C. nivalis* | UTEX SNO71 | LC360465 | LC360470 | LC360484 | LC360488 | LC360492 |
|  | Hakkoda-Green^5^ | LC438436^6^ | LC438440^6^ | LC438444^6^ | LC438448^6^ | LC438452^6^ |
|  | Tateyama-Green^5^ | LC438437^6^ | LC438441^6^ | LC438445^6^ | LC438449^6^ | LC438453^6^ |
|  | Tateyama-Orange^5^ | LC438438^6^ | LC438442^6^ | LC438446^6^ | LC438450^6^ | LC438454^6^ |
|  | Gassan-B^1^ | LC012714 | LC060478 | LC012722 | LC012730 | LC012743 |
|  | Gassan-C^1^ | LC012715 | LC060479 | LC012723 | LC012731 | LC012744 |
|  | Gassan-NIV1^7^ |  |  |  |  | AB434274 |
|  | Gassan-NIV2^7^ |  |  |  |  | AB434275 |
|  | Hakkoda-3^2^ | LC012716 | LC060480 | LC012724 | LC012732 | LC012745 |
|  | P24/DR4^8^ |  |  |  |  | KY499616 |
| *C. nivalis* subsp. *tatrae* | LP01^9^ |  |  |  |  | KY499615 |
| *C. pichinchae* | UTEX SNO33 | AB906346 | LC060481 | AB906365 | AB906376 | AB434266,  LC012746 |
| *C. remiasii* | CCCryo^10^ 005-99  (authentic) | LC360466 | LC360471 | LC360485 | LC360489 | LC360493 |
| *C. tenuis* | UTEX SNO132  (authentic) | AB906347 | AB906355 | AB906366 | AB906377 | AB434263 |
| *C. tughillensis* | UTEX SNO91  (authentic) | AB906348 | AB906356 | AB906367 | AB906378 | LC012747 |
| Outgroup | | | | | | |
| *C. asteroidea* | SAG^11^ 11-47^12^ | U70783 | LC360473 | AB084808 | AB084342 | AB022225 |
| *C. augustae* | SAG 5.73^12^ | AJ410452 | LC360474 | AB504757 | AB504769 | AB504764 |
| *C. chlorococcoides* | SAG 15.82  (authentic) | AJ410449,  AB624555 | AB906359 | AB624580 | AB624595 | LC361432 |
| *C. kasaiae* | NIES-2862  (authentic) | AB734109 | LC360475 | AB734110 | AB734111 | LC012751 |
| *C. pseudoplatyrhyncha* | NIES-2563 | AB548689 | LC360476 | AB548690 | AB548691 | LC012752 |
| *C. radiata* | UTEX 966^12^ | U57697 | LC360477 | AB084311 | AB084345 | AJ001878 |
| *C. reticulata* | SAG 29.83  (= UTEX 1970^12^) | U70791,  AB624560 | AF395508 | AB084312 | AB084346, AB084347 | AB022534 |
| *C. serbinowii* | UTEX 492^12^  (= SAG 11.84) | U70795, AB624568, AB624569 | LC360478 | AB084317 | AB084354 | AJ001879 |
| *C. typhlos* | SAG 26.86  (= UTEX 1969) | AB624566 | LC360479 | AB084307 | AB084341 | AB022228 |
| *G. lateperforata* | NIES-464 | AB504779 | LC360480 | AB504761 | AB504773 | AB504768 |
| *G. rubrifilum* | SAG 3.85^13^ | AJ410455 | LC360481 | AB504758 | AB504770 | AB504765 |
| *I. deasonii* | SAG 46.72  (authentic) | AJ410446 | LC360482 | AB101503 | AB101514 | AB101508 |
| *I. pauromitos* | NIES-3707  (authentic) | LC057290 | LC360483 | LC360487 | LC360491 | LC360495 |

Abbreviations: *atp*B, ATP synthase beta subunit gene; *C.*, *Chloromonas*; *G.*, *Gloeomonas*; *I.*, *Ixipapillifera*; LSU rDNA, the large subunit of ribosomal DNA; *psa*B, P700 chlorophyll *a* apoprotein A2 gene; *rbc*L, the large subunit of the RuBisCO gene; SSU rDNA, the small subunit of ribosomal DNA.

^1^Specimen of zygotes collected from snowpack on Mt. Gassan, Japan [1].

^2^Specimen of zygotes collected from snowpack on Mt. Hakkoda, Japan [1].

^3^Culture Collection of Algae at the University of Texas at Austin [2,3].

^4^Microbial Culture Collection at the National Institute for Environmental Studies [4,5].

^5^See S1 Table.

^6^Sequenced in the present study.

^7^Specimen of zygotes collected from snowpack on Mt. Gassan, Japan [6].

^8^Specimen of zygotes collected from snowpack on Austrian Alps [7,8].

^9^Specimen of zygotes collected from snowpack in Slovakia [8].

^10^Culture Collection of Cryophilic Algae at the Fraunhofer Institute for Cell Therapy and Immunology [9].

^11^Sammlung von Algenkulturen at the University of Göttingen [10,11].

^12^Epitype proposed by Pröschold et al. [12].

^13^Epitype of *Chloromonas rubrifilum* designated by Pröschold et al. [12].

**References**

1. Matsuzaki R, Kawai-Toyooka H, Hara Y, Nozaki H. Revisiting the taxonomic significance of aplanozygote morphologies of two cosmopolitan snow species of the genus *Chloromonas* (Volvocales, Chlorophyceae). Phycologia. 2015;54: 491–502. doi: 10.2216/15-33.1.

2. Starr RC, Zeikus JA. UTEX – the culture collection of algae at the University of Texas at Austin. J Phycol. 1993;29 Suppl 2: 1–106. doi: 10.1111/j.0022-3646.1993.00001.x.

3. UTEX Culture Collection of Algae [Internet]. Texas: The University of Texas at Austin; c2017 [cited 2018 Oct 17]. Available from: <https://utex.org/>.

4. Kawachi M, Ishimoto M, Mori F, Yumoto K, Sato M, Noël M-H. MCC-NIES. List of Strains, 9th Edition [DVD]. Tsukuba: National Institute for Environmental Studies; 2013.

5. Microbial Culture Collection at National Institute for Environmental Studies [Internet]. Ibaraki: The National Institute for Environmental Studies; c2001 [cited 2018 Oct 17]. Available from: <http://mcc.nies.go.jp/index_en.html>.

6. Muramoto K, Kato S, Shitara T, Hara Y, Nozaki H. Morphological and genetic variation in the cosmopolitan snow alga *Chloromonas nivalis* (Volvocales, Chlorophyceae) from Japanese mountainous area. Cytologia. 2008;73: 91–96. doi: 10.1508/cytologia.73.91.

7. Remias D, Karsten U, Lütz C, Leya T. Physiological and morphological processes in the alpine snow alga *Chloromonas nivalis* (Chlorophyceae) during cyst formation. Protoplasma. 2010;243: 73–86. doi: 10.1007/s00709-010-0123-y. PubMed PMID: 20229328.

8. Procházková L, Remias D, Řezanka T, Nedbalová L. *Chloromonas nivalis* subsp. *tatrae*, subsp. nov. (Chlamydomonadales, Chlorophyta): re-examination of a snow alga from the High Tatra Mountains (Slovakia). Fottea. 2018;18: 1–18. doi: 10.5507/fot.2017.010.

9. Culture Collection of Cryophilic Algae [Internet]. Brandenburg: The Fraunhofer Institute for Cell Therapy and Immunology; c2016 [cited 2018 Oct 17]. Available from: <http://cccryo.fraunhofer.de/web/infos/welcome/>.

10. Schlösser UG. SAG – Sammlung von Algenkulturen at the University of Göttingen. Catalogue of strains 1994. Bot Acta. 1994;107: 113–186. doi: 10.1111/j.1438-8677.1994.tb00784.x.

11. The Culture Collection of Algae at the University of Göttingen [Internet]. Göttingen: The University of Göttingen; c2017 [cited 2018 Oct 17]. Available from: <http://sagdb.uni-goettingen.de/index.php>.

12. Pröschold T, Marin B, Schlösser UG, Melkonian M. Molecular phylogeny and taxonomic revision of *Chlamydomonas* (Chlorophyta). I. Emendation of *Chlamydomonas* Ehrenberg and *Chloromonas* Gobi, and description of *Oogamochlamys* gen. nov. and *Lobochlamys* gen. nov. Protist. 2001;152: 265–300. doi: 10.1078/1434-4610-00068. PubMed PMID: 11822658.
