# Supplementary material for: Taxonomic re-examination of “Chloromonas nivalis (Volvocales, Chlorophyceae) zygotes” from Japan and description of C. muramotoi sp. nov
Source: PLoS One. 2019 Jan 24;14(1):e0210986. doi: 10.1371/journal.pone.0210986 (PMC6345437; doi:10.1371/journal.pone.0210986)
Supplement: S4 Table — (DOCX) [file pone.0210986.s010.docx]

**S4 Table. Substitution models applied to the respective data matrices of the present phylogenetic analyses (Figs 2 and 3).**

|  | Phylogenetic method | | |
| --- | --- | --- | --- |
| Data matrix/Figure No. | BI | ML | NJ |
| concatenated SSU and LSU  rDNA, *atp*B and *psa*B (first,  second codons only) from  29 OTUs/Fig 2 | partitioned into  SSU rDNA (GTR+I+G),  LSU rDNA (GTR+I+G),  *atp*B (SYM+I+G), and  *psa*B (GTR+I+G)^1^ | partitioned into  SSU rDNA (K2P+I),  LSU rDNA (GTR+G4),  *atp*B (TIM2e+I), and  *psa*B (TNe+I)^2^ | TrN+I+G^3^ |
| *rbc*L from 34 OTUs/Fig 3 | partitioned into  first codons (GTR+I+G),  second codons (JC+G), and  third codons (GTR+I+G)^1^ | partitioned into  first codons (TIM+G4),  second codons (JC+I), and  third codons (TPM3u+G4)^2^ | GTR+I+G^3^ |

Each substation model was selected by hierarchical likelihood ratio test using MrModeltest 2.3 [1] for Bayesian inference (BI) or Modeltest 3.7 [2] for neighbor-joining (NJ) analysis, or selected by the Bayesian information criterion using IQ-TREE v. 1.4.3 [3] for maximum likelihood (ML) analysis.

Abbreviations: *atp*B, ATP synthase beta subunit gene; LSU rDNA, the large subunit of ribosomal DNA; OTUs, operational taxonomic units; *psa*B, P700 chlorophyll *a* apoprotein A2 gene; *rbc*L, the large subunit of the RuBisCO gene; SSU rDNA, the small subunit of ribosomal DNA.

**References**

1. Nylander JAA. MrModeltest 2.3 [software]. 2008 May 22 [cited 2018 Oct 17]. Available from: <https://github.com/nylander/MrModeltest2>.

2. Posada D, Crandall KA. Modeltest: testing the model DNA substitution. Bioinformatics. 1998;14: 817–818. doi: 10.1093/bioinformatics/14.9.817. PubMed PMID: 9918953.

3. Nguyen L-T, Schmidt HA, von Haeseler A, Minh BQ. IQ-TREE: A fast and effective stochastic algorithm for estimating maximum likelihood phylogenies. Mol Biol Evol. 2015;32: 268–274. doi: 10.1093/molbev/msu300. PubMed PMID: 25371430; PubMed Central PMCID: PMC4271533.
