# Supplementary material for: Taxonomic re-examination of “Chloromonas nivalis (Volvocales, Chlorophyceae) zygotes” from Japan and description of C. muramotoi sp. nov
Source: PLoS One. 2019 Jan 24;14(1):e0210986. doi: 10.1371/journal.pone.0210986 (PMC6345437; doi:10.1371/journal.pone.0210986)
Supplement: S5 Table — (DOCX) [file pone.0210986.s011.docx]

**S5 Table. Morphological characteristics of field-collected “*Chloromonas nivalis* zygotes” examined by scanning electron microscopy.**

| Origin | Number of flanges | Flange form | Bifurcated flanges | Cell width ×  cell length (μm) | Reference |
| --- | --- | --- | --- | --- | --- |
| USA | 6–8 | Each flange is straight or spiraled (rarely crenulate or rippled); many flanges extend to the entire or almost entire length of the cell | not observed | 10–21 × 16–37 | [1] |
| Norway (Svalbard) | 6–8 | Each flange is straight or spiraled (rarely crenulate or rippled); flanges do not extend to the entire length of the cell | observed | 14.3±2.3 × 26.9±3 | [2] |
| Norway (Svalbard) | − | Each flange is straight, undulant or spiraled; many flanges extend to the entire or almost entire length of the cell | not observed | − | [3] |
| Austria | − | Each flange is spiraled; many flanges extend to the entire or almost entire length of the cell | not observed | − | [4] |
| Japan^1^ | several | All flanges on the cell are uniform, straight or sigmate; many flanges extend to the entire or almost entire length of the cell; in straight flanges, short ones reaching to neither pole also occur | not observed | 11–15 × 20–28 | [5] |
| Austria^2^ | 8–9 | Each flange is undulant; not all of them extend to the entire length of the cell; short flanges reaching to neither pole also occur | observed | 12.6±1.3 × 21.5±1.6 | [6] |
| Japan^3^ | 8–10 | Each flange is straight or slightly undulant; not all of them extend to the entire length of the cell; flanges reaching to neither pole also occur | not observed | 10–16 × 20–26 | [7]; present study |
| Slovakia^4^ | 10–12 | Each flange is straight or slightly undulant; many flanges extend to the entire length of the cell or from one pole nearly to the antapex; short flanges reaching neither pole also occur | observed | 12.8±0.9 × 19.8±1.3 | [8] |
| Japan^5^ | almost 8 | Each flange is straight or slightly undulant; four long flanges reach to the entire length of the cell, whereas the remaining short flanges are medially located and extend to neither pole; each short flange is positioned between two long flanges | not observed | 9.1–13.4 × 15.6–22.4 | present study |

^1^Cells were used for a specimen Gassan-NIV1 or Gassan-NIV2.

^2^Cells (collected on 19 Aug 2004) were used for a specimen P24/DR4.

^3^Cells were used for a specimen Gassan-C, which is considered conspecific with *C. miwae*.

^4^Cells were used for a specimen of the zygotes of *C. nivalis* subsp. *tatrae*, LP01.

^5^Cells were used for three specimens Hakkoda-Green, Tateyama-Green and Tateyama-Orange.

**References**

1. Hoham RW, Mullet JE. The life history and ecology of the snow alga *Chloromonas cryophila* sp. nov. (Chlorophyta, Volvocales). Phycologia. 1977;16: 53–68. doi: 10.2216/i0031-8884-16-1-53.1.

2. Müller T, Bleiß W, Martin C-D, Rogaschewski S, Fuhr G. Snow algae from northwest Svalbard: their identification, distribution, pigment and nutrient content. Polar Biol. 1998;20: 14–32. doi: 10.1007/s003000050272.

3. Leya T. Feldstudien und genetische Untersuchungen zur Kryophilie der Schneealgen Nordwestspitzbergens [dissertation]. Berlin: Humboldt-Universität zu Berlin; 2004. German.

4. Holzinger A, Lütz C. Algae and UV irradiation: Effort on ultrastructure and related metabolic functions. Micron. 2006;37: 190–207. doi: 10.1016/j.micron.2005.10.015. PubMed PMID: 16376552.

5. Muramoto K, Kato S, Shitara T, Hara Y, Nozaki H. Morphological and genetic variation in the cosmopolitan snow alga *Chloromonas nivalis* (Volvocales, Chlorophyta) from Japanese mountainous area. Cytologia. 2008;73: 91–96. doi: 10.1508/cytologia.73.91.

6. Remias D, Karsten U, Lütz C, Leya T. Physiological and morphological processes in the Alpine snow alga *Chloromonas nivalis* (Chlorophyceae) during cyst formation. Protoplasma. 2010;243: 73–86. doi: 10.1007/s00709-010-0123-y. PubMed PMID: 20229328.

7. Matsuzaki R, Kawai-Toyooka H, Hara Y, Nozaki H. Revisiting the taxonomic significance of aplanozygote morphologies of two cosmopolitan snow species of the genus *Chloromonas* (Volvocales, Chlorophyceae). Phycologia. 2015;54: 491–502. doi: 10.2216/15-33.1.

8. Procházková L, Remias D, Řezanka T, Nedbalová L. *Chloromonas nivalis* subsp. *tatrae*, subsp. nov. (Chlamydomonadales, Chlorophyta): re-examination of a snow alga from the High Tatra Mountains (Slovakia). Fottea. 2018;18: 1–18. doi: 10.5507/fot.2017.010.
